# Supplementary material for: Recognition and reporting of suspected adverse drug reactions by surveyed healthcare professionals in Uganda: key determinants
Source: BMJ Open. 2014 Nov 24;4(11):e005869. doi: 10.1136/bmjopen-2014-005869 (PMC4244492; doi:10.1136/bmjopen-2014-005869)
Supplement: Author's manuscript [file bmjopen-2014-005869.draft_revisions.pdf]

BMJ Open

# Recognition and reporting of suspected adverse drug reactions by surveyed healthcare professionals in Uganda: key determinants

For peer review only

| | |
|---|---|
| Journal: | *BMJ Open* |
| Manuscript ID: | bmjopen-2014-005869 |
| Article Type: | Research |
| Date Submitted by the Author: | 05-Jun-2014 |
| Complete List of Authors: | Kiguba, Ronlad; Makerere University College of Health Sciences, Pharmacology and Therapeutics<br>Karamagi, Charles; Makerere University College of Health Sciences, Clinical Epidemiology Unit, Department of Medicine<br>Waako, Paul; Makerere University College of Health Sciences, Pharmacology and Therapeutics<br>Ndagije, Helen; National Drug Authority, National Pharmacovigilance Centre<br>Bird, Sheila; MRC Biostatistics Unit, |
| <b>Primary Subject Heading</b>: | Health services research |
| Secondary Subject Heading: | Epidemiology, Health policy, Medical education and training, Pharmacology and therapeutics |
| Keywords: | pharmacoviligance, adverse drug reactions, reporting-rates, Uganda, healthcare professionals |

SCHOLARONE™
Manuscripts

# Recognition and reporting of suspected adverse drug reactions by surveyed healthcare professionals in Uganda: key determinants

*Ronald Kiguba[1]*, Charles Karamagi[2], Paul Waako[1], Helen B. Ndagije[3] and Sheila M. Bird[4]*

[1]Department of Pharmacology and Therapeutics, Makerere University College of Health Sciences, Kampala, Uganda

[2]Clinical Epidemiology Unit, Department of Medicine, Makerere University College of Health Sciences, Kampala, Uganda

[3]National Pharmacovigilance Centre, National Drug Authority, Kampala, Uganda

[4]Medical Research Council Biostatistics Unit, Cambridge, United Kingdom

* Corresponding author

Email addresses:

RK: kiguba@gmail.com

CK: ckaramagi2000@yahoo.com

PW: pwaako@chs.mak.ac.ug

HBN: hndagije@gmail.com

SMB: sheila.bird@mrc-bsu.cam.ac.uk

For peer review only



For peer review only - http://bmjopen.bmj.com/site/about/guidelines.xhtml

## ABSTRACT

**Objective**

To assess extent and determinants of past-month recognition of suspected adverse drug reactions (ADR) and past-year ADR reporting among healthcare professionals (HCPs) in Uganda.

**Setting**

Geographically diverse health facilities (public, private for-profit, private not-for-profit).

**Participants**

1,345 HCPs, two-thirds of those to whom questionnaire was distributed.

**Primary and secondary outcome measures**

Percent HCPs who suspected ADR in the past-month; reported ADR in the past-year.

**Results**

Nurses were the majority (58%, 776/1,340). Only half the respondents had heard about pharmacovigilance: 39% of nurses (295/763; 95% CI: 35% to 42%), 70% otherwise (383/547; 95% CI: 66% to 74%). One fifth (268/1,289 or 21%; 95% CI: 19% to 23%) had suspected an ADR in the previous 4 weeks, 111 of them nurses; 15% (190/1,296) had reported a suspected ADR in the past-year, 103 of them nurses.

Past-month ADR suspicion was more likely by non-nurses (odds ratio (OR) = 1.7, 95% CI: 1.16 – 2.40) and with medical research involvement (OR = 1.5, 95% CI: 1.05 – 2.15) but past-month receipt of patient ADR-complaint predominated (OR = 19, 95% CI: 14-28).



For peer review only - http://bmjopen.bmj.com/site/about/guidelines.xhtml

Past-year ADR reporting was higher by hospital staff (OR = 1.9, 95% CI: 1.18-3.10), especially in medicine (OR = 2.3, 95% CI: 1.08-4.73); but lower from private for-profit health facilities (OR = 0.5, 95% CI: 0.28-0.77) and by older staff (OR = 0.6, 95% CI: 0.43-0.91); more likely by HCPs who had ever encountered a fatal ADR (OR = 2.9, 95% CI: 1.94-4.25), knew to whom to report (OR = 1.7, 95% CI: 1.18-2.46), or suggested how to improve ADR reporting (OR = 1.6, 95% CI: 1.04-2.49). Two attitudinal factors were important: diffidence and lethargy.

**Conclusions**

One in five HCPs suspected an ADR in the past-month. Empowering patients could strengthen ADR detection and reporting in Africa.

## Strengths

- Over 1300 healthcare professionals surveyed in diverse health-facilities in Uganda
- Return-rate of self-completion questionnaire was two-thirds
- Attitudes to pharmacovigilance elicited
- Demographic and professional determinants ascertained of past-month ADR suspicion and past-year ADR reporting.

## Limitations

- Purposely-selected survey locations
- Non-random sampling of healthcare professionals
- Self-report as the main method of inquiry
- Temporal relationship between past-year ADR reporting and some explanatory factors (patient-ADR-complaint in the past-month) could not be determined
- Under-representation of nurses



For peer review only - http://bmjopen.bmj.com/site/about/guidelines.xhtml

- Several respondents may have referred to the same suspected ADR but this did not have a significant bearing since our main focus is assessment of individual ADR reporting behaviour rather than individual ADRs.

For peer review only



For peer review only - http://bmjopen.bmj.com/site/about/guidelines.xhtml

# Recognition and reporting of suspected adverse drug reactions by surveyed healthcare professionals in Uganda: key determinants

*Ronald Kiguba[1], Charles Karamagi[2], Paul Waako[1], Helen B. Ndagije[3] and Sheila M. Bird[4]*

[1]Department of Pharmacology and Therapeutics, Makerere University College of Health Sciences, Kampala, Uganda

[2]Clinical Epidemiology Unit, Department of Medicine, Makerere University College of Health Sciences, Kampala, Uganda

[3]National Pharmacovigilance Centre, National Drug Authority, Kampala, Uganda

[4]Medical Research Council Biostatistics Unit, Cambridge, United Kingdom

For peer review only

## Background

Adverse drug reactions (ADRs) are significant causes of patient morbidity and mortality[1] and are known to raise overall healthcare costs[2-5]. The World Health Organization (WHO) defines pharmacovigilance (PV) as "the science and activities relating to the detection, assessment, understanding and prevention of adverse effects or any other medicine-related problem"[6]. Spontaneous and voluntary reporting of suspected ADRs generates signals about rare, delayed and unexpected drug reactions that are undetected in the initial phases of drug development[7] but under-reporting is a major limitation[8]. Studies conducted elsewhere have estimated that only 6-10% of all ADRs are reported[9-11]. This low rate of ADR reporting undermines efforts to identify and estimate the magnitude of drug risks, confirmation of actionable issues, and possible regulatory action[12].



For peer review only - http://bmjopen.bmj.com/site/about/guidelines.xhtml

Widespread use of electronic medical record databases has enhanced patient safety through automation of signal detections for ADRs, thereby improving healthcare service delivery[13]. In Africa, the establishment and use of such databases is still rare [14] and ADR reporting is largely done manually. Strengthening of PV systems in sub-Saharan African (SSA) countries has received support from global health initiatives, but reporting is often siloed by disease (e.g. malaria, vaccines, HIV/AIDS) because of restricted funding streams rather than strengthening countrywide reporting systems[15]. As a result, PV systems in SSA remain weak[16]. In Uganda, 556 spontaneous reports were submitted to the National Pharmacovigilance Centre (NPC) in the initial five years of 2005-2009. Of these, 315 (57%) were related to medicines with 10 or more spontaneous ADR reports and were dominated by antiretroviral drugs (51%, 160/315), antimalarials (27%, 85/315), and antibiotics (22%, 70/315)[17]. The dominance of ADR reports related to these groups of medicines accords with the burden of disease in SSA[18].

The WHO's Uppsala Monitoring Centre (UMS) maintains web-based ADR reporting software (VigiFlow) for use by National Pharmacovigilance Centres[19]. Although receipt of 200 or more ADR reports per million population per year is desirable[20], most SSA countries submit fewer than 20 ADR reports per million population in 2010 compared to more than 100 reports per million in other low- and middle-income countries[21].

Uganda established a National Pharmacovigilance Centre (NPC) in 2005 and has been a member of the WHO program for International Drug Monitoring since 2007. In 2010, there was a training-of-trainers session for 30 national pharmacovigilance trainers. By 2011, 14 regional PV centres were established[21], PV-training sessions for core teams of healthcare professionals (HCPs) were conducted in



For peer review only - http://bmjopen.bmj.com/site/about/guidelines.xhtml

each of these centres, and ADR reporting forms distributed[22]. At least one support supervision visit per centre is conducted annually. Despite these efforts, reporting rate in Uganda (population: 36 million) is still low at 6 ADR reports per million population per year, based on 1,348 ADR reports in 2007-2012 [180, 75, 229[23], 140, 183, 413 in 2012 (when Targeted Spontaneous Reporting (TSR) was launched); and 128 in January-June 2013 (Nassali Huldah & Helen Ndagije, personal communication, 15 Jan 2014)]. Moreover, significant missing information in four-fifths of ADR reports compromises analysis[17].

Of 46 SSA countries whose PV systems were assessed to determine their capacity to ensure drug safety, Uganda was identified as one of four with active PV systems that could, in principle, detect, evaluate, and address medicine safety issues[24]. Indeed, Ugandan surgical series[25] on, and subsequent media coverage of, gluteal fibrosis and post injection paralysis among children injected with quinine[26, 27] triggered investigation by the Ugandan NPC which, in 2010, mediated change of Uganda's recommended quinine injection site from the gluteus muscle to the thigh[28].

Personal and professional characteristics associated with increased ADR reporting by HCPs include older age, male gender, lower workload, higher number of prescriptions issued per day, type of education received, specific PV training, and involvement in teaching and research[8, 29, 30]. Inhibitory factors include: unavailability of ADR forms, bureaucratic method of ADR reporting, and uncertainty over which professional cadre is mandated to report ADRs[31].

In 1996, Inman et al[32] described eight 'deadly sins' to explain why HCPs underreport ADRs: i) attitudes related to professional activities (financial incentives, fear of litigation, and ambition to publish personal case series), ii) ADR–related knowledge and attitudes (complacency, diffidence, indifference, and ignorance), and



For peer review only - http://bmjopen.bmj.com/site/about/guidelines.xhtml

iii) excuses made by HCPs (lethargy). Insecurity is an attitudinal factor that was not proposed by Inman but has been reported elsewhere[33].

In Africa, there is a paucity of empirical data on PV awareness[34-38]. Hence we sought to determine the level of PV awareness by HCPs, and the extent and determinants of past-month ADR recognition and of past-year ADR reporting in Uganda.

## Methods

### Study design and sampling procedure

From 25 May 2012 through 28 February 2013, we conducted a survey across Uganda in purposively selected, geographically diverse public and private health facilities. Public institutions included the National Referral Hospital-Mulago, and six Regional Referral Hospitals. In addition, we included District Hospitals and Health Centres (HCs) at levels II to IV in the catchment area where a Regional Referral Hospital was selected. For logistical reasons, we selected a convenience sample of private for-profit and private not-for-profit health facilities (which included drug shops) in the respective districts where public institutions were assessed. Permission to conduct the research was sought from the administrators of the selected institutions.

Any HCP involved in prescribing, transcribing, dispensing medication orders, and administration of drugs to a hospital inpatient was eligible for inclusion. Written informed consent was obtained from HCPs prior to their recruitment. The self-completed questionnaires did not contain identifying information on individual HCPs. The survey team used serial numbers to track distributed questionnaires. Five research assistants, all final year medical students at Mulago National Referral Hospital, were initially recruited, trained on the concepts of pharmacovigilance, informed consent



For peer review only - http://bmjopen.bmj.com/site/about/guidelines.xhtml

and response-rate and on the survey by self-completing it in an average of 27 minutes (22, 25, 27, 31, 31). A similar model of data collection by pre-trained investigators was employed in the upcountry sites.

Given the challenge of accessing staff lists in the selected health facilities (and especially so in private-for-profit settings), random sampling of eligible HCPs was not practicable. Instead, in each health facility, the pre-trained investigators approached HCPs of all ranks and invited them to complete a pretested questionnaire, of which 2,200 were printed and 2,000 distributed. Invitations might be declined if HCPs were particularly busy or, despite willingness, a delay of several days or weeks might ensue before the self-completion questionnaire was returned. In practice, neither the refusal-rate by approached HCPs nor the 'did not return rate' for distributed questionnaires was reliably documented.

In Uganda, there were reckoned to be 46,566 HCPs in 2009[39], who would have been survey-eligible had they worked at the survey-locations. Doctors and dentists (3,459) represented an estimated 7% of the nationally eligible staff but were 20% of the achieved sample; 762 pharmacists and pharmacy technicians 1.6% of nationally eligible staff but 6% of the achieved sample; and 37,625 nurses, midwives and nursing assistants an estimated 81% of the nationally eligible staff but 59% of the achieved sample.

**Data collection and management**

The survey questionnaire, see **Appendix**, elicited demographic and professional information, description of the most recent suspected ADR, and attitudes to, as well as knowledge and use of, the suspected ADR reporting system. The questionnaire for HCPs included 15 attitudinal statements on ADR reporting to be



For peer review only - http://bmjopen.bmj.com/site/about/guidelines.xhtml

scored from 1 (total disagreement) to 5 (total agreement). All data were entered into a databank using EpiData 3.1.

The questionnaire was initially tested on 125 participants. The subsequent revisions sufficiently minor that results of the pre-test were included in the final analysis.

**Statistical analysis**

Responses are summarized as frequencies and percentages. Different potential determinants for the past-month recognition or past-year reporting of suspected ADRs were screened using $\chi^2$-tests for categorical variables. Logistic regression was then used to assess the relationship of demographic and professional factors severally to: i) recognition of suspected ADRs in the past 4 weeks; and for those in post for at least one year, ii) having reported at least one suspected ADR in the past 12 months. Attitudinal factors were also incorporated in ii). Missing data were accounted for using multiple imputations under the missing at random assumption[40] on the one hand or, as here, the missing-assigned approach on the other, where missing data were meaningfully assigned to an existing category. Results are expressed as odds ratios (ORs) with 95% confidence intervals. Statistical analyses were carried out using Stata 12.0[41].

**Ethical clearance**

Ethical approval was obtained from the School of Medicine Research and Ethics Committee, Makerere University College of Health Sciences, and the Uganda National Council for Science and Technology.



For peer review only - http://bmjopen.bmj.com/site/about/guidelines.xhtml

# Results

## Study population

Of 2,000 questionnaires distributed, 1,345 were completed, a return-rate of 67%. Mean age of respondent HCPs was 32.4 years (SD = 8.9). Nurses were the majority (776/1,340 or 58%), see **Table 1**.

## Awareness of pharmacovigilance

Half the respondents (678/1,310 or 52%; 95% CI: 49% to 55%) had ever heard about pharmacovigilance: two-fifths of nurses (295/763 or 39%; 95% CI: 35% to 42%) but 70% of others (383/547; 95% CI: 66% to 74%). Thirty percent of HCPs (412/1,317; 95% CI: 29% to 34%) were aware of the existence of Uganda's NPC but only 3% (37/1,312; 95% CI: 2% to 4%) of HCPs had *ever* submitted an ADR report to the NPC.

## Suspected ADR reporting in the previous 12 months

Only 15% of HCPs (190/1,296; 95% CI: 13% to 17%) had reported a suspected ADR in the previous 12 months, of whom 15% (27/175) claimed to have made their report to NPC so that our respondents' past-year ADR reporting rate to NPC was an estimated 1 in 50 (2%). Only 41% (11/27; 95% CI: 22% to 59%) past-year reporters to NPC had found the NPC-form clear on what to report.

When HCPs were asked about when, in the past 12 months, they had reported their most recent suspected ADR, 79/178 (44%) said within the past month, 28 (16%) in the months 2+3 prior, and 71 (40%) in months 4-12, a distribution indicative either of a multiplicity of reports per ADR-reporter or biased recall.



For peer review only - http://bmjopen.bmj.com/site/about/guidelines.xhtml

**ADR recognition**

Twenty one per cent (268/1,289: 95% CI, 19% to 23%) of respondents had suspected an ADR in the previous one month, 76% of whom (195/257: 95% CI, 71% to 81%) had received patient ADR-complaints in the past month. Of HCPs who had suspected an ADR in the past month, 35% (92/262: 95% CI, 29% to 41%) had reported an ADR in the past 12 months.

Among HCPs who had not suspected an ADR in the previous month, 12% (121/1,000: 95% CI, 10% to 14%) had nonetheless received patient ADR-complaints in the past month.

In the previous 4 weeks, see **Table 2**, 26% (340/1,302) of HCPs had received 1,190 patient ADR-complaints [mean of 3.5 complaints (sd 9.5) per complaint-receiving HCP] which equates to 0.9 ADR-complaints (95% CI: 0.65 to 1.18) per HCP per month. Also, 21% (268/1,289) of HCPs had suspected 670 ADRs [mean of 2.5 suspected ADRs (sd 2.6) per suspecting HCP] which equates to 0.5 suspected ADRs (95% CI: 0.45 to 0.59) per HCP per month, implying an ADR suspicion rate of 0.57 (0.52/0.91) per patient ADR-complaint per HCP per month (95% CI: 0.42 to 0.80).

Among the 15% (190/1,296) who were ADR-reporters in the previous 12 months, 44% (79/178) claimed to have submitted their most recent report in the past 4 weeks. If so, there could be at least 84 suspected ADR reports submitted by 1,296 HCPs in the past 4 weeks (or 0.065 ADR-reports in past 4 weeks per HCP) when 0.5 ADRs were suspected in the past 4 weeks per HCP. This translates into a 13% ADR-report rate per suspected ADR.



For peer review only - http://bmjopen.bmj.com/site/about/guidelines.xhtml

**Medication classes and fatalities in survey-described suspected ADRs**

The most frequently mentioned medication classes associated with 182 survey-described ADRs in the past 4-weeks which cited one or more drugs (216 drug citations) were antibiotics (38%, 83/216), antiretroviral agents (23%, 49/216), antimalarials (15%, 33/216, 15 of which implicated quinine), analgesics (9%, 19/216), and others (15%, 32/216).

Two suspected ADRs were described by HCPs which involved child fatalities in association with quinine: a 5-year-old girl had been given intravenous quinine and died soon after arrival at a private-not-for-profit hospital in Eastern Uganda; and a 2-year old boy had reacted to quinine and died despite the doctor in a public hospital in Eastern Uganda having administered an antidote. Full details of HCPs' described suspected ADRs will be reported separately.

**Feedback to ADR reporters**

Reporters of ADRs to AIDS Treatment Information Centre (ATIC) received the highest feedback (60%, 12/20), followed by those who reported to the Medical Superintendent or Institutional Review Board (39%: 23/58 + 4/11). Feedback from Uganda's NPC was infrequent (23%: 5/22). Reporters of ADRs to drug manufacturers (4) or District Directors of Health Services (12) received zero feedback.

**Reasons for ADR reporting**

The commonest reason that respondents vouched for ADR reporting was that the patient had developed a serious ADR (30%, 48/159 reasons) followed by patient safety (18%, 29/159), and patient ADR-complaint (8%, 13/159). The next three reasons each had nine citations: institutional mandate to report ADRs, prevention of similar ADRs, and as a means of obtaining advice.


For peer review only - http://bmjopen.bmj.com/site/about/guidelines.xhtml
For peer review only

**Attitudes to ADR reporting**

Only 14% (186/1,301: 95% CI, 12% to 16%) of respondents indicated that reporting ADRs put their career at risk, see **Table 3**, while 36% (466/1,304: 95% CI, 33% to 38%) thought that it is only necessary to report serious or unexpected ADRs. Most respondents agreed that they have a professional obligation to report ADRs (76%, 1,000/1,311: 95% CI, 74% to 79%) and 68% (896/1,319: 95% CI, 65% to 70%) stated that they would report ADRs if there were an easier method. Forty five per cent (596/1,312: 95% CI, 43% to 48%) stated that they do not know how information reported in the ADR form is used, 64% (833/1,309: 95% CI, 61% to 66%) felt that they would report an ADR only if they were sure it was related to use of a particular drug, and 27% (349/1,305: 95% CI, 24% to 29%) felt that they should be financially reimbursed for providing the ADR reporting service.

**Factors associated with ADR suspicion in the past month**

Suspicion of ADR in the past 4-weeks was more likely by non-nurses (odds ratio (OR) = 1.7, 95% CI: 1.16 – 2.40) and with involvement in medical research (OR = 1.5, 95% CI: 1.05 – 2.15) but the clearly dominant factor was that the HCP had received patient ADR-complaint(s) in the past 4-weeks (OR = 19, 95% CI: 14-28). There was some evidence that ADR suspicion was less likely by staff in surgical wards, see **Table 4**.

Logistic regression analysis among the 973 respondents who did not receive a patient ADR complaint did not identify any additional significant cofactors associated with ADR suspicion.


For peer review only - http://bmjopen.bmj.com/site/about/guidelines.xhtml

**Personal, professional and attitudinal factors associated with having made an ADR report in the past 12 months**

Demographic and professional factors associated with a lower likelihood to report ADRs in the past 12 months were: private for-profit health facility (vs. public; OR = 0.5, 95% CI: 0.28 – 0.77) and HCP aged over 30 years (OR = 0.6, 95% CI: 0.43 – 0.91); while those associated with being more likely to report ADRs included: medical department (vs. surgery; OR = 2.3, 95% CI: 1.08 – 4.73), having ever encountered a fatal ADR (OR = 2.9, 95% CI: 1.94 – 4.25), knowing to whom to report ADRs (OR = 1.7, 95% CI: 1.18 – 2.46), and HCPs who had suggested ways of improved ADR reporting (OR = 1.6, 95% CI: 1.04 – 2.49), see **Table 5.**

Only two attitudinal factors were additionally relevant: diffidence ('the belief that reporting an ADR would only be done if there was certainty that it was related to the use of a particular drug'; OR = 0.6, 95% CI: 0.41-0.89) and lethargy ('I do not know how information reported in ADR form is used'), see **Table 6**.

**Suggestions for improved ADR reporting**

The most frequently cited suggestion was to sensitize, train and provide ongoing medical education on ADRs to HCPs (42%, 667/1,589 suggestions) followed by making ADR forms available (17%, 262/1,589), sensitizing the public and counselling patients about ADRs (11%, 166/1,589), creating a coordinating office in each health facility (5%, 73/1,589), providing financial incentives to reporters (4%, 65/1,589), and making available telephone or online ADR reporting systems (4%, 57/1,589), see **Table 7**.



For peer review only - http://bmjopen.bmj.com/site/about/guidelines.xhtml

For peer review only

## Discussion

A low proportion of HCPs reported having submitted an adverse drug reaction (ADR) report in the previous 12 months (15%) and the level of awareness of PV was also low, similar to observations made elsewhere[34, 42, 43]. Healthcare professionals from different cadres may recognize suspected ADRs but fail to take the responsibility to report[44]. Barely one in eight (13%) of suspected ADRs in the past month was reported by the HCPs in that same period, yet around three-fifths of patient ADR-complaints in the past month were adjudged by HCPs to be suspected ADRs. Integration of pharmacovigilance into pre-service training curricula and emphasizing its importance in promoting patient safety in healthcare delivery is a first step[45, 46] upon which other PV initiatives can build.

To raise the number of submitted ADR reports, Uganda has proposed mandatory reporting of ADRs by industry and HCPs[22]. However, questions have been raised about the effectiveness of compulsory reporting by HCPs[47] and the NPC needs to improve its feedback to ADR reporters since our respondents ranked it much lower than ATIC. Moreover, HCPs in our study reported ADRs to a greater extent than in nationally-reported statistics: 2% of HCPs (27/1281: 95% CI, 1.3% to 2.9%) had reported any suspected ADR to the NPC in the previous year compared with the NPC's annual average national ADR reporting rate for Uganda from 2007 to mid-2013 of 0.44% [based on 1,348 reports in 6.5 years from 46,566 clinical staff countrywide: 95% CI, 0.38% to 0.51%] or 0.90% in the highest report-year of 2012 [413 reports in 2012: 95% CI, 0.80% to 0.97%]. Thus, HCPs in our study seemed at least twice as likely to have submitted suspected ADRs to the NPC in the previous year when compared with the national ADR reporting rates by Uganda's HCPs.



For peer review only - http://bmjopen.bmj.com/site/about/guidelines.xhtml

One limitation to our estimates is that more than one HCP may have described (and reported) the same suspected ADR since our ability to discriminate between suspected ADRs was compromised by variation in the quality of ADR descriptions, a limitation that NPC also contends with.

Consistent with ADR reports from the NPC[17], we identified antibiotics, antiretroviral agents, and antimalarials as the three most frequently cited medication classes in survey-described ADRs. Therefore, health initiatives already focusing on the PV of these medications, if replicated for other classes, present opportunities to strengthen overall PV systems in these settings[17]. As a PV exemplar in Uganda, the NPC and AIDS Control Program introduced TSR in 2011 to monitor tenofovir for renal toxicity and to detect suspected ADRs related to antiretroviral therapy use in the Prevention of Mother to Child Transmission of HIV and in the Early Infants Diagnosis program[48]. Results from TSR are yet to be disseminated, however.

Around three-fifths of patients' ADR-complaints to HCPs in the past month translated into ADR suspicion. Patient ADR-complaint was dominant among explanatory factors for HCPs' ADR-suspicion in the past month and so we suggest that empowering patients to support HCPs may improve the detection and reporting of suspected ADRs. Moreover, other countries have instituted systems that promote spontaneous direct patient reporting of suspected ADRs thus permitting patients to participate in PV activities that teach them to handle their medicines better and improves their communication with HCPs[49, 50].

Improvement of the ADR reporting form for Uganda seems necessary. Therefore, our research team designed a form that is relevant to the inpatient setting and captures additional information required for causality assessment of suspected medicines. This form will be tested in a follow-up study on inpatients.



For peer review only - http://bmjopen.bmj.com/site/about/guidelines.xhtml

Other suggestions to improve ADR reporting by respondents included; increased visibility of the NPC and giving useful feedback to ADR reporters, introducing telephone and online reporting systems, increasing onsite support supervision, making ADR forms more available, providing training and continued medical education of HCPs, and sensitizing the public to ADRs. The absence of a national PV policy, however, coupled with the lack of proper coordination between the NPC and numerous health programmes and sentinel sites may undermine efforts to strengthen the countrywide PV system[17]. For example, in Uganda's teaching hospitals, could some clinical grand rounds address PV and suspected serious ADRs?

Although previous studies suggested a positive relationship between older age and ADR reporting[51, 52], we found that older HCPs ($\geq$30 years) were less likely than their younger counterparts to have reported suspected ADRs in the past 12 months. These contrasting results might be attributed to idiosyncratic differences between HCPs and healthcare systems in Europe and Africa such that younger staff, as in our study, may have had more PV training. There is, as yet, limited published literature from other African settings. Our respondents were, on average, 10 years younger when compared with studies conducted in Europe[29]. We suggest that older HCPs in Uganda be targeted in future strategies on improved ADR reporting.

In contrast to other studies[52], training on how to report ADRs was not significantly associated with increased ADR reporting. Given the cross-sectional study design we used, it was not possible to establish whether PV training preceded ADR reporting, or vice versa, and therefore we were unable to assess their temporal relationship. That notwithstanding, Gonzalez-Gonzalez *et al* have suggested that multifaceted interventions, as opposed to single educational programmes, increase to a greater extent HCPs' PV awareness and motivate them to report ADRs[8].



For peer review only - http://bmjopen.bmj.com/site/about/guidelines.xhtml

A low level of PV awareness may lead to underreporting of ADRs[53]. In our study, knowing to whom to report was an important factor for ADR reporting in the final logistic regression. We also observed that the proportion (31%: 95% CI, 29% to 34%) of respondents aware of the existence of Uganda's NPC is lower than reported for Nigeria [52% (51/99): 95% CI, 42% to 61%][34]. Much higher proportions of PV awareness have been reported in Europe[29] and Asia[54, 55] where there are higher ADR reporting rates per million of population[56] and more government involvement in national PV programs[34].

Healthcare professionals who had ever encountered a fatal ADR were twice as likely to report an ADR as HCPs who had not. Correspondingly, development of a serious or fatal ADR was the most frequently cited reason for ADR reporting. We also found that HCPs who suggested possible ways of improving the ADR reporting system were more likely to have reported an ADR in the previous 12 months[57].

Healthcare professionals who agreed with the statement 'I would only report an ADR if I was sure that it was related to the use of a particular drug' (diffidence) were less likely to report suspected ADRs. Apart from diffidence and lethargy/indifference ('I do not know how information reported in the ADR form is used'), none of the other Inman factors was associated with ADR reporting[8, 32, 58]. Diffidence and lethargy can be targeted in educational interventions to promote ADR reporting and by improved feedback to ADR-reporters.

Although provision of financial incentives to reporters was the fifth most frequently cited suggestion to improve ADR reporting, it was not statistically significant in the logistic regression for the odds on ADR reporting and these findings are consistent with those in the developed world[59].



For peer review only - http://bmjopen.bmj.com/site/about/guidelines.xhtml

For peer review only

In private for-profit health facilities, HCPs were less likely to have reported ADRs in the previous 12 months than their counterparts in the public sector. In addition, HCPs in hospitals (public and private) were twice as likely as those from other health facilities (HCs II & III, community pharmacies, drug shops) to have reported suspected ADRs in the previous 12 months. Whereas few PV scale-up activities in Africa have given priority to the private sector[16, 22], more public-private collaboration could strengthen PV systems in our SSA setting[60].

Our study had several limitations. First, we used self-report as the main method of inquiry and this may have introduced recall bias. Second, we may have experienced social desirability bias as HCPs may not have given frank responses for fear of being embarrassed if they were not reporting ADRs. However, as we used self-administered questionnaires without respondents' names, the potential for this bias was reduced. Third, the cross-sectional design that we used could not establish temporal relationships between ADR reporting in past year and some explanatory factors. Fourth, there was over-representation of doctors and pharmacists/pharmacy technicians versus nurses. Finally, several respondents may have referred to the same suspected ADR but this did not have a significant bearing since our main focus was assessment of individual ADR reporting behaviour rather than on individual ADRs.

Our study has, however, generated key insights on determinants in Uganda for HCPs' ADR suspicion and reporting.

## Conclusions

One in five HCPs had suspected an ADR in the past 4 weeks while one in seven had reported an ADR in the previous 12 months. Empowering patients to support HCPs in suspected ADR detection and reporting is essential to strengthening PV systems in Africa. HCPs who ever encountered fatal ADRs are keener reporters



For peer review only - http://bmjopen.bmj.com/site/about/guidelines.xhtml

and can consequently help others to avoid the experience that made them better reporters. HCPs ought to know that they don't have to be certain about causality to report suspected ADRs. Poor access to suspected ADR forms and lack of feedback on reports are constraints that can be rectified. [4,500 words]


## Acknowledgements

The authors wish to thank all the HCPs who agreed to participate in this study.

We also thank Huldah Nassali at the National Pharmacovigilance Centre for providing technical support to this project.

Finally, RK thanks Noeline Nakasujja at Makerere University College of Health Sciences, and Yukari C. Manabe at Johns Hopkins University for supporting his development of the first three drafts of the manuscript.

## Funding

This work was supported by Training Health Researchers into Vocational Excellence (THRiVE) in East Africa, grant number 087540, funded by the Wellcome Trust; grant number 5R24TW008886 supported by OGAC, NIH and HRSA; and an African Doctoral Dissertation Research Fellowship award offered by the African Population and Health Research Centre (APHRC) in partnership with the International Development Research Centre (IDRC). In UK, SMB is funded by Medical Research Council programme number MC_U105260794.

## Role of funders

The work here reported is solely the responsibility of the authors and does not necessarily represent the official views of the supporting offices.

The funders had no role in the decisions on what and where to publish.




For peer review only - http://bmjopen.bmj.com/site/about/guidelines.xhtml

## Competing Interests

SMB holds GSK shares. The authors declare that they otherwise have no competing interests.

## Authors' contributions

RK conceived of the study and drafted the manuscript and, in conjunction with SMB, participated in its design, implementation, statistical analysis and the drawing of inferences. CK, PW and HBN participated in study design and in the process of manuscript writing. All authors approved the final manuscript.

**Data Sharing Statement**

Data for categorical questions in the survey are available on request from the lead author.

For peer review only



For peer review only - http://bmjopen.bmj.com/site/about/guidelines.xhtml

References

1. Lazarou J, Pomeranz BH, Corey PN. Incidence of adverse drug reactions in hospitalized patients: a meta-analysis of prospective studies. *JAMA* 1998; 279(15): 1200-5.

2. Ramesh M, Pandit J, Parthasarathi G. Adverse drug reactions in a south Indian hospital--their severity and cost involved. *Pharmacoepidemiol Drug Saf* 2003; 12(8): 687-92.

3. Classen DC, Pestotnik SL, Evans RS, Lloyd JF, Burke JP. Adverse drug events in hospitalized patients. Excess length of stay, extra costs, and attributable mortality. *JAMA* 1997; 277(4): 301-6.

4. Bates DW, Spell N, Cullen DJ, et al. The costs of adverse drug events in hospitalized patients. Adverse Drug Events Prevention Study Group. *JAMA* 1997; 277(4): 307-11.

5. Gautier S, Bachelet H, Bordet R, Caron J. The cost of adverse drug reactions. *Expert Opin Pharmacother* 2003; 4(3): 319-26.

6. World Health Organization. The Safety of Medicines in Public Health Programmes: Pharmacovigilance an Essential Tool. 2006. http://www.who.int/medicines/areas/quality_safety/safety_efficacy/Pharmacovigilance_B.pdf (accessed 31 May 2014).

7. Lexchin J. Is there still a role for spontaneous reporting of adverse drug reactions? *CMAJ : Canadian Medical Association journal = journal de l'Association medicale canadienne* 2006; 174(2): 191-2.

8. Lopez-Gonzalez E, Herdeiro MT, Figueiras A. Determinants of under-reporting of adverse drug reactions: a systematic review. *Drug Saf* 2009; 32(1): 19-31.

9. Hazell L, Shakir SA. Under-reporting of adverse drug reactions : a systematic review. *Drug Saf* 2006; 29(5): 385-96.

10. Smith CC, Bennett PM, Pearce HM, et al. Adverse drug reactions in a hospital general medical unit meriting notification to the Committee on Safety of Medicines. *Br J Clin Pharmacol* 1996; 42(4): 423-9.

11. Pirmohamed M, Breckenridge AM, Kitteringham NR, Park BK. Adverse drug reactions. *BMJ* 1998; 316(7140): 1295-8.



For peer review only

For peer review only - http://bmjopen.bmj.com/site/about/guidelines.xhtml

12. Gavaza P, Brown CM, Lawson KA, Rascati KL, Wilson JP, Steinhardt M. Influence of attitudes on pharmacists' intention to report serious adverse drug events to the Food and Drug Administration. *Br J Clin Pharmacol* 2011; 72(1): 143-52.

13. Miguel A, Azevedo LF, Lopes F, Freitas A, Pereira AC. Methodologies for the detection of adverse drug reactions: comparison of hospital databases, chart review and spontaneous reporting. *Pharmacoepidemiol Drug Saf* 2013; 22(1): 98-102.

14. Alj L, Touzani MDW, Benkirane R, Edwards IR, Soulaymani R. Detecting medication errors in pharmacovigilance database: Capacities and limits. *Int J Risk Saf Med* 2007; 19: 187-94.

15. Strengthening Pharmaceutical Systems (SPS). Supporting Pharmacovigilance in Developing Countries: The Systems Perspective
Arlington, VA: Submitted to the U.S. Agency for International Development by the SPS Program, 2009.

16. Isah AO, Pal SN, Olsson S, Dodoo A, Bencheikh RS. Specific features of medicines safety and pharmacovigilance in Africa. *Therapeutic Advances in Drug Safety* 2012; 3(1): 25-34.

17. National Pharmacovigilance Centre. Working together with the Public Health Programmes: a regulator's perspective for addressing the minimum requirements for Pharmacovigilance. 2010.
http://www.who.int/medicines/areas/quality_safety/regulation_legislation/icdra/WG-2_2Dec.pdf (accessed 21 June 2013).

18. Institute for Health Metrics and Evaluation. The Global Burden of Disease: Generating Evidence, Guiding Policy-Sub-Saharan Africa Regional Edition. 2013. http://www.healthdata.org/policy-report/global-burden-disease-generating-evidence-guiding-policy%E2%80%94sub-saharan-africa-regional (accessed 31 May 2014).

19. Uppsala Monitoring Centre (UMC). VigiFlow ™ The complete Individual Case Safety Report (ICSR) management system from the UMC. http://www.who-umc.org/graphics/27705.pdf (accessed 31 May 2014).

20. Uppsala Monitoring Centre (UMC). Pharmacovigilance - Reporting Trends. 2013. http://who-umc.org/DynPage.aspx?id=108476&mn1=7347&mn2=7252&mn3=7322&mn4=7558 (accessed 31 May 2014).

21. Strengthening Pharmaceutical Systems (SPS) Program. Safety of Medicines in sub-Saharan Africa: Assessment of Pharmacovigilance Systems and their



For peer review only - http://bmjopen.bmj.com/site/about/guidelines.xhtml

Performance. 2011.
http://apps.who.int/medicinedocs/documents/s19152en/s19152en.pdf2014).

22. Ouma C, Abwao E. Africa Pharmacovigilance Meeting 2012: Technical Report. Submitted to the US Agency for International Development by the Systems for Improved Access to Pharmaceuticals and Services Program, 2012.

23. National Pharmacovigilance Centre. The Pharmacovigilance News. 2010. http://www.nda.or.ug/docs/bulletin%20issue%20No.1.pdf (accessed 1 June 2014).

24. Appiah B. Africa struggles to improve drug safety. *CMAJ : Canadian Medical Association journal = journal de l'Association medicale canadienne* 2012; 184(10): E533-4.

25. Ekure J. Gluteal Fibrosis. A report of 28 cases from Kumi Hospital, Uganda. *East and Central African Journal of Surgery* 2006; 12(1): 144-7.

26. Ekure J. Post-injection paralysis: The new polio in Uganda. 9 December 2007. http://www.newvision.co.ug/D/9/34/601055.

27. Chinnock P. Concern in Uganda over children injured by poorly administered malaria treatment 2009. http://www.tropika.net/svc/news/20090604/Chinnock-20090604-News-Quinine-Uganda (accessed 13 May 2014).

28. National Pharmacovigilance Centre. What is current practice of Pharmacovigilance in Uganda? 2012.
http://africapv2012.files.wordpress.com/2012/04/day-2_5_country-presentations-nras_h-ngadije-uganda-compatibility-mode.pdf (accessed 11 December 2013).

29. Bateman DN, Sanders GL, Rawlins MD. Attitudes to adverse drug reaction reporting in the Northern Region. *Br J Clin Pharmacol* 1992; 34(5): 421-6.

30. Figueiras A, Tato F, Fontainas J, Gestal-Otero JJ. Influence of physicians' attitudes on reporting adverse drug events: a case-control study. *Med Care* 1999; 37(8): 809-14.

31. Rawlins MD. Pharmacovigilance: paradise lost, regained or postponed? The William Withering Lecture 1994. *J R Coll Physicians Lond* 1995; 29(1): 41-9.

32. Inman WH. Attitudes to adverse drug reaction reporting. *Br J Clin Pharmacol* 1996; 41(5): 434-5.

33. Herdeiro MT, Figueiras A, Polonia J, Gestal-Otero JJ. Physicians' attitudes and adverse drug reaction reporting : a case-control study in Portugal. *Drug Saf* 2005; 28(9): 825-33.

For peer review only



For peer review only - http://bmjopen.bmj.com/site/about/guidelines.xhtml

34. Oshikoya KA, Awobusuyi JO. Perceptions of doctors to adverse drug reaction reporting in a teaching hospital in Lagos, Nigeria. *BMC Clin Pharmacol* 2009; 9: 14.

35. Robins AH, Weir M, Biersteker EM. Attitudes to adverse drug reactions and their reporting among medical practitioners. *South African medical journal = Suid-Afrikaanse tydskrif vir geneeskunde* 1987; 72(2): 131-4.

36. Ball D, Tisocki T. Adverse drug reaction reporting by general medical practitioners and retail pharmacists in Harare--a pilot study. *Cent Afr J Med* 1998; 44(8): 190-5.

37. Okezie EO, Olufunmilayo F. Adverse drug reactions reporting by physicians in Ibadan, Nigeria. *Pharmacoepidemiol Drug Saf* 2008; 17(5): 517-22.

38. Jaquet A, Djima MM, Coffie P, et al. Pharmacovigilance for antiretroviral drugs in Africa: lessons from a study in Abidjan, Cote d'Ivoire. *Pharmacoepidemiol Drug Saf* 2011; 20(12): 1303-10.

39. Africa Health Workforce Observatory. Human Resources for Health Country Profile - Uganda. 2009. p. 30.

40. Sterne JA, White IR, Carlin JB, et al. Multiple imputation for missing data in epidemiological and clinical research: potential and pitfalls. *BMJ* 2009; 338: b2393.

41. StataCorp. Stata Statistical Software: Release 12. College Station, TX: StataCorp LP; 2011.

42. Khan SA, Goyal C, Chandel N, Rafi M. Knowledge, attitudes, and practice of doctors to adverse drug reaction reporting in a teaching hospital in India: An observational study. *Journal of natural science, biology, and medicine* 2013; 4(1): 191-6.

43. Pimpalkhute SA, Jaiswal KM, Sontakke SD, Bajait CS, Gaikwad A. Evaluation of awareness about pharmacovigilance and adverse drug reaction monitoring in resident doctors of a tertiary care teaching hospital. *Indian journal of medical sciences* 2012; 66(3-4): 55-61.

44. Bemt van den PMLA, Egberts ACG. Drug-related problems: definitions and classifications. *EJHP* 2007; (13): 62-4.

45. Gallagher TH, Waterman AD, Ebers AG, Fraser VJ, Levinson W. Patients' and physicians' attitudes regarding the disclosure of medical errors. *JAMA* 2003; 289(8): 1001-7.

For peer review only



For peer review only - http://bmjopen.bmj.com/site/about/guidelines.xhtml

46. Cox AR, Marriott JF, Wilson KA, Ferner RE. Adverse drug reaction teaching in UK undergraduate medical and pharmacy programmes. *J Clin Pharm Ther* 2004; 29(1): 31-5.

47. Cross C. Drug reporting may be mandatory for doctors. *CMAJ : Canadian Medical Association journal = journal de l'Association medicale canadienne* 2013; 185(10): 866.

48. Uppsala Monitoring Centre. Uppsala Reports 59. 2012.

49. Andrew Herxheimer, Rose Crombag, Teresa Leonardo Alves. Direct Patient Reporting of Adverse Drug Reactions. May 2010.

50. Masso Guijarro P, Aranaz Andres JM, Mira JJ, Perdiguero E, Aibar C. Adverse events in hospitals: the patient's point of view. *Quality & safety in health care* 2010; 19(2): 144-7.

51. Tubert-Bitter P, Haramburu F, Begaud B, Chaslerie A, Abraham E, Hagry C. Spontaneous reporting of adverse drug reactions: who reports and what? *Pharmacoepidemiol Drug Saf* 1998; 7(5): 323-9.

52. Irujo M, Beitia G, Bes-Rastrollo M, Figueiras A, Hernandez-Diaz S, Lasheras B. Factors that influence under-reporting of suspected adverse drug reactions among community pharmacists in a Spanish region. *Drug Saf* 2007; 30(11): 1073-82.

53. Ohaju-Obodo JO, Iribhogbe OI. Extent of pharmacovigilance among resident doctors in Edo and Lagos states of Nigeria. *Pharmacoepidemiol Drug Saf* 2010; 19(2): 191-5.

54. Li Q, Zhang SM, Chen HT, et al. Awareness and attitudes of healthcare professionals in Wuhan, China to the reporting of adverse drug reactions. *Chin Med J (Engl)* 2004; 117(6): 856-61.

55. Chatterjee S, Lyle N, Ghosh S. A survey of the knowledge, attitude and practice of adverse drug reaction reporting by clinicians in Eastern India. *Drug Saf* 2006; 29(7): 641-2.

56. Aagaard L, Strandell J, Melskens L, Petersen PS, Holme Hansen E. Global patterns of adverse drug reactions over a decade: analyses of spontaneous reports to VigiBase. *Drug Saf* 2012; 35(12): 1171-82.

57. Passier A, ten Napel M, van Grootheest K, van Puijenbroek E. Reporting of adverse drug reactions by general practitioners: a questionnaire-based study in the Netherlands. *Drug Saf* 2009; 32(10): 851-8.



For peer review only - http://bmjopen.bmj.com/site/about/guidelines.xhtml

58. Herdeiro MT, Figueiras A, Polonia J, Gestal-Otero JJ. Influence of pharmacists' attitudes on adverse drug reaction reporting : a case-control study in Portugal. *Drug Saf* 2006; 29(4): 331-40.

59. Herdeiro MT, Polonia J, Gestal-Otero JJ, Figueiras A. Factors that influence spontaneous reporting of adverse drug reactions: a model centralized in the medical professional. *J Eval Clin Pract* 2004; 10(4): 483-9.

60. World Health Organization (WHO) - MMV joint technical consultation on active pharmacovigilance monitoring with a special focus on affordable medicine facility for malaria (AMFm). Pharmacovigilance Guidance for Countries Participating in AMGm Phase 1. 2009. http://apps.who.int/medicinedocs/documents/s18427en/s18427en.pdf (accessed 1 June 2014).

For peer review only



For peer review only - http://bmjopen.bmj.com/site/about/guidelines.xhtml

**Table 1: Demographic and professional characteristics of healthcare professionals, Uganda, 2013**

| | | |
|---|---|---|
| **Total number of participants** | | **1,345** |
| **Age, n = 1,253** | | |
| Mean years (SD); median, inter-quartile range | 32.4 ( 8.9); 30, 26-36 | |
| **Gender, n = 1,345** | | |
| Male | | 541 (40.2) |
| Female | | 804 (59.8) |
| **Number of Patients seen per day, n = 1,226** | | |
| Mean number (SD); median, inter-quartile range | 41.0 (46.3); 30, 15-50 | |
| **Professional Cadre, n = 1,340** | | |
| Nurse | | 792 (59.1) |
| Doctor | | 275 (20.5) |
| Pharmacist & Pharmacy Technician | | 84 ( 6.3) |
| Other | | 44 ( 3.3) |
| **Type of Health Facility, n = 1,345** | | |
| Public | | 568 (42.2) |
| Private Not-For-Profit | | 280 (20.8) |
| Private-For-Profit | | 497 (37.0) |
| **Highest Academic Qualification, n = 1,345** | | |
| Certificate | | 471 (35.0) |
| Diploma | | 501 (37.3) |
| First Degree | | 294 (21.9) |
| Masters Degree or PhD | | 79 ( 5.9) |
| **Ever received ADR training, n = 1,225** | | |
| Yes | | 180 (14.7) |
| No | | 1045 (85.3) |
| **Received Patient ADR Complaint in past 4 weeks, n = 1,302** | | |
| Yes | | 340 (26.1) |
| No | | 962 (73.9) |

For peer review only

Table 2: Patient ADR complaints and healthcare professional ADR suspicion in past 4 weeks, Uganda, 2013

| Patient ADR-Complaints/Healthcare Professional ADR suspicion | | | | | |
|---|---|---|---|---|---|
| **Patient ADR-Complaints in past 4 weeks** | | | | | |
| Cadre | No of HCPs | Who received complaints | Mean (SD) ADR-Complaints | ADR-complaints received | ADR-complaints per HCP |
| Overall | 1,302 | 340 (26%) | 3.5 ( 9.5) | 1,190 | 0.91 |
| **Nurses** | **760** | **155 (20%)** | **3.9 (11.4)** | **604** | **0.80** |
| **Non-nurses** | **542** | **185 (34%)** | **3.2 ( 7.7)** | **592** | **1.09** |
| Doctors | 270 | 97 (36%) | 3.3 (10.2) | 320 | 1.19 |
| Pharm/Ptech | 81 | 34 (42%) | 3.9 ( 4.0) | 132 | 1.64 |
| Other | 191 | 54 (28%) | 2.5 ( 2.1) | 135 | 0.71 |
| **Healthcare Professionals' ADR suspicion in past 4 weeks** | | | | | |
| Cadre | No of HCPs | Who suspected ADRs | Mean (SD) suspected ADRs | ADR Suspicions by HCPs | ADR-suspicion per HCP |
| Overall | 1,289 | 268 (21%) | 2.5 (2.6) | 670 | 0.52 |
| **Nurses** | **756** | **111 (15%)** | **2.6 (2.6)** | **288** | **0.38** |
| **Non-nurses** | **533** | **157 (29%)** | **2.5 (2.6)** | **393** | **0.74** |
| Doctors | 267 | 88 (33%) | 2.3 (2.5) | 202 | 0.76 |
| Pharm/Ptech | 80 | 23 (29%) | 2.9 (3.2) | 66 | 0.83 |
| Other | 186 | 46 (25%) | 2.5 (2.5) | 114 | 0.61 |

For peer review only

**Table 3: Healthcare professionals' responses to 15 attitudinal statements on ADR reporting, Uganda, 2013**

| Statement | Agree | Neutral | Disagree |
|---|---|---|---|
| Serious ADRs are well documented by the time a drug is marketed | 820 (61.7) | 166 (12.5) | 343 (25.8) |
| It is nearly impossible to determine whether a drug is responsible for a particular adverse reaction | 527 (39.8) | 189 (14.3) | 607 (45.9) |
| I would only report an ADR if I was sure that it was related to the use of a particular drug | 833 (63.6) | 138 (10.6) | 338 (25.8) |
| The one case of an ADR that an individual health worker might see makes no significant contribution to medical knowledge | 210 (16.2) | 122 (9.4) | 966 (74.4) |
| I read articles about adverse drug reactions with interest | 824 (63.3) | 180 (13.8) | 298 (22.9) |
| I have a professional obligation to report ADRs | 1000 (76.3) | 143 (10.9) | 168 (12.8) |
| Reporting ADRs puts my career at risk | 186 (14.3) | 126 (9.7) | 989 (76.0) |
| It is only necessary to report serious or unexpected ADRs | 466 (35.7) | 129 (9.9) | 709 (54.4) |
| I do not have time to complete an ADR report form | 143 (10.9) | 208 (15.8) | 963 (73.3) |
| I do not have the time to actively look for ADRs while at work | 195 (14.8) | 152 (11.6) | 968 (73.6) |
| I do not know how information reported in ADR form is used | 596 (45.4) | 194 (14.8) | 522 (39.8) |
| I talk with pharmaceutical companies about possible ADRs with their drugs | 290 (22.2) | 202 (15.5) | 813 (62.3) |
| I think the best way to report ADRs is by publishing in medical literature | 701 (53.4) | 238 (18.1) | 374 (28.5) |
| I should be financially reimbursed for providing the ADR service | 349 (26.7) | 199 (15.3) | 757 (58.0) |
| I would be more likely to report ADRs if there were an easier method | 896 (67.9) | 169 (12.8) | 254 (19.3) |

For peer review only

**Table 4: Personal and professional factors associated with ADR suspicion in the past 4 weeks among 1,289 healthcare professionals, Uganda, 2013**

| Factor | ADR Suspicion | | Crude Analysis | | | Adjusted Analysis | | |
|---|---|---|---|---|---|---|---|---|
| | Yes (%) | No (%) | OR | 95%CI | P-value | OR | 95%CI | P-value |
| Level of Health Facility | | | | | | | | |
| Other | 77 (16.1) | 413 (83.9) | 1.0 | | | 1.0 | | |
| Hospital | 191 (23.5) | 621 (76.5) | 1.6 | 1.19-2.14 | 0.002 | 1.3 | 0.81-2.06 | 0.286 |
| Type of Health Facility | | | | | | | | |
| Public | 129 (23.2) | 426 (76.8) | 1.0 | | | 1.0 | | |
| Private Not-For-Profit | 55 (20.5) | 213 (79.5) | 0.9 | 0.60-1.22 | 0.380 | 0.8 | 0.51-1.27 | 0.353 |
| Private For-Profit | 84 (18.0) | 382 (82.0) | 0.7 | 0.53-0.99 | 0.041 | 0.8 | 0.49-1.30 | 0.362 |
| Region of the country | | | | | | | | |
| Central | 148 (25.3) | 437 (74.7) | 1.0 | | | 1.0 | | |
| Eastern | 62 (15.1) | 348 (84.9) | 0.5 | 0.38-0.73 | <0.001 | 0.6 | 0.37-0.94 | 0.025 |
| Other | 58 (19.7) | 236 (80.3) | 0.7 | 0.52-1.02 | 0.066 | 0.8 | 0.50-1.22 | 0.270 |
| **Professional Cadre** | | | | | | | | |
| Nurse | 111 (14.7) | 645 (85.3) | 1.0 | | | 1.0 | | |
| Non-nurse | 157 (29.5) | 376 (70.5) | 2.4 | 1.84-3.19 | <0.001 | 1.7 | 1.16-2.40 | 0.005 |
| Age | | | | | | | | |
| Less than 30 years | 119 (20.8) | 452 (79.2) | 1.0 | | | 1.0 | | |
| Aged 30 years or older | 149 (20.8) | 569 (70.3) | 1.0 | 0.76-1.30 | 0.969 | 0.9 | 0.65-1.31 | 0.647 |
| Patient Load | | | | | | | | |
| Greater than 30/day | 128 (22.2) | 449 (77.8) | 1.0 | | | 1.0 | | |
| At most 30/day | 140 (19.7) | 572 (80.3) | 0.9 | 0.66-1.12 | 0.268 | 1.2 | 0.85-1.75 | 0.272 |
| Department | | | | | | | | |
| Surgery | 13 (13/1) | 86 (86.9) | 1.0 | | | 1.0 | | |
| Medicine | 150 (23.7) | 482 (76.3) | 2.1 | 1.12-3.79 | 0.021 | 2.1 | 0.99-4.38 | 0.054 |
| Paediatrics, Obs&Gyn | 40 (20.2) | 158 (79.8) | 1.7 | 0.85-3.30 | 0.136 | 2.0 | 0.90-4.57 | 0.090 |
| Other | 65 (18.1) | 295 (81.9) | 1.5 | 0.77-2.77 | 0.250 | 1.4 | 0.66-3.18 | 0.358 |
| **Involved in medical research** | | | | | | | | |
| No | 160 (17.6) | 749 (82.3) | 1.0 | | | 1.0 | | |
| Yes | 108 (38.6) | 272 (61.4) | 1.9 | 1.40-2.46 | <0.001 | 1.5 | 1.05-2.15 | 0.026 |
| Ever encountered Fatal ADR | | | | | | | | |
| No | 197 (19.0) | 842 (81.0) | 1.0 | | | 1.0 | | |
| Yes | 71 (28.4) | 179 (71.6) | 1.7 | 1.24-2.32 | 0.001 | 1.1 | 0.71-1.64 | 0.732 |
| Knowing to whom to report | | | | | | | | |
| No | 129 (20.2) | 511 (79.8) | 1.0 | | | 1.0 | | |
| Yes | 139 (21.4) | 510 (78.6) | 1.1 | 0.82-1.41 | 0.577 | 1.2 | 0.86-1.74 | 0.254 |
| Suggestions for improved ADR reporting | | | | | | | | |
| No | 54 (17.0) | 264 (83.0) | 1.0 | | | 1.0 | | |
| Yes | 214 (22.0) | 757 (78.0) | 1.4 | 0.99-1.92 | 0.054 | 0.9 | 0.60-1.37 | 0.628 |

For peer review only

| | | | | | | | | |
|---|---|---|---|---|---|---|---|---|
| **Received patient ADR complaint in past 4 weeks** | | | | | | | | |
| No | 73 ( 7.5) | 900 (92.5) | 1.0 | | | 1.0 | | |
| Yes | 195 (61.7) | 121 (38.3) | 19.9 | 14.3-27.6 | <0.001 | 19.0 | 13.5-27.1 | <0.001 |

For peer review only

**Table 5: Personal and professional factors associated with ADR reporting in the past 12 months among 1,164 healthcare professionals who had been in post for at least 1 year, Uganda, 2013**

| Factor | ADR Reporter | | Crude Analysis | | | Adjusted Analysis | | |
|---|---|---|---|---|---|---|---|---|
| | Yes (%) | No (%) | OR | 95%CI | P-value | OR | 95%CI | P-value |
| Level of Health Facility | | | | | | | | |
| Other | 36 (8.0) | 413 (92.0) | 1.0 | | | 1.0 | | |
| Hospital | 128 (17.9) | 587 (82.1) | 2.5 | 1.69-3.70 | <0.001 | 1.9 | 1.18-3.10 | 0.008 |
| Type of Health Facility | | | | | | | | |
| Public | 91 (18.5) | 402 (81.5) | 1.0 | | | 1.0 | | |
| Private Not-For-Profit | 40 (16.8) | 198 (83.2) | 0.9 | 0.59-1.34 | 0.585 | 0.8 | 0.50-1.23 | 0.286 |
| Private For-Profit | 33 (7.6) | 400 (92.4) | 0.4 | 0.24-0.56 | <0.001 | 0.5 | 0.28-0.77 | 0.003 |
| Region of the country | | | | | | | | |
| Central | 82 (15.9) | 433 (84.1) | 1.0 | | | 1.0 | | |
| Eastern | 36 (9.7) | 334 (90.3) | 0.6 | 0.38-0.86 | 0.008 | 0.7 | 0.43-1.13 | 0.140 |
| Other | 46 (16.5) | 233 (83.5) | 1.0 | 0.70-1.55 | 0.836 | 1.2 | 0.75-1.84 | 0.471 |
| Professional Cadre | | | | | | | | |
| Nurse | 93 (13.5) | 597 (86.5) | 1.0 | | | 1.0 | | |
| Non-nurse | 71 (15.0) | 403 (85.0) | 1.1 | 0.81-1.58 | 0.470 | 0.8 | 0.55-1.18 | 0.264 |
| Age | | | | | | | | |
| Less than 30 years | 70 (15.0) | 396 (85.0) | 1.0 | | | 1.0 | | |
| Aged 30 years or older | 94 (13.5) | 604 (86.5) | 0.9 | 0.63-1.23 | 0.455 | 0.6 | 0.43-0.91 | 0.014 |
| Patient Load | | | | | | | | |
| Greater than 30/day | 84 (16.1) | 439 (83.9) | 1.0 | | | 1.0 | | |
| At most 30/day | 80 (12.5) | 561 (87.5) | 0.7 | 0.54-1.04 | 0.081 | 0.9 | 0.61-1.27 | 0.510 |
| Department | | | | | | | | |
| Surgery | 10 (11.5) | 77 (88.5) | 1.0 | | | 1.0 | | |
| Medicine | 95 (16.3) | 488 (83.7) | 1.5 | 0.75-3.00 | 0.253 | 2.3 | 1.08-4.73 | 0.030 |
| Paediatrics, Obs&Gyn | 18 (10.5) | 153 (89.5) | 0.9 | 0.40-2.06 | 0.065 | 0.8 | 0.36-1.95 | 0.675 |
| Other | 41 (12.7) | 282 (87.3) | 1.1 | 0.54-2.34 | 0.147 | 1.6 | 0.73-3.50 | 0.243 |
| Involved in medical research | | | | | | | | |
| No | 103 (12.6) | 716 (87.4) | 1.0 | | | 1.0 | | |
| Yes | 61 (17.7) | 284 (82.3) | 1.5 | 1.06-2.11 | 0.023 | 1.3 | 0.88-1.87 | 0.191 |
| Ever encountered Fatal ADR | | | | | | | | |
| No | 98 (10.7) | 820 (89.3) | 1.0 | | | 1.0 | | |
| Yes | 62 (27.1) | 167 (72.9) | 3.0 | 2.12-4.33 | <0.001 | 2.9 | 1.94-4.25 | <0.001 |
| Knowing to whom to report | | | | | | | | |
| No | 62 (11.0) | 504 (89.1) | 1.0 | | | 1.0 | | |
| Yes | 102 (17.1) | 496 (82.9) | 1.7 | 1.19-2.35 | 0.003 | 1.7 | 1.18-2.46 | 0.005 |
| Suggestions for improved ADR reporting | | | | | | | | |
| No | 32 (10.6) | 270 (89.4) | 1.0 | | | 1.0 | | |
| Yes | 132 (15.3) | 730 (84.7) | 1.5 | 1.01-2.30 | 0.044 | 1.6 | 1.04-2.49 | 0.032 |

For peer review only

**Table 6: Attitudinal factors associated with ADR reporting in past 12 months among 1,114 healthcare professionals who responded to attitudinal questions, Uganda, 2013**

| Factor | Reported an ADR in the past 12 months | | Crude Analysis | | | Adjusted Analysis* | | |
|---|---|---|---|---|---|---|---|---|
| | Yes (%) | No (%) | OR | 95% CI | P-value | OR | 95% CI | P-value |
| I do not know how information reported in ADR form is used | | | | | | | | |
| Agree | 64 (12.5) | 447 (87.5) | 0.7 | 0.47-0.97 | 0.031 | 0.7 | 0.46-1.00 | 0.052 |
| Neutral | 17 (10.6) | 143 (89.4) | 0.6 | 0.32-0.98 | 0.041 | 0.5 | 0.27-0.94 | 0.030 |
| Disagree | 81 (17.5) | 383 (82.5) | 1.0 | | | 1.0 | | |
| I would only report an ADR if I was sure that it was related to the use of a particular drug | | | | | | | | |
| Agree | 86 (12.2) | 620 (87.8) | 0.6 | 0.39-0.81 | 0.002 | 0.6 | 0.41-0.89 | 0.011 |
| Neutral | 12 (9.9) | 109 (90.1) | 0.4 | 0.23-0.87 | 0.015 | 0.6 | 0.29-1.17 | 0.128 |
| Disagree | 60 (19.7) | 244 (80.3) | 1.0 | | | 1.0 | | |

*Adjusted for personal and professional characteristics: level of health facility, type of health facility, region, non-nurse as professional cadre, age, patient load, department, involvement in medical research, ever encountered a fatal ADR, knowing to whom to report ADRs, and suggesting ways to improve ADR reporting

For peer review only

**Table 7: Suggested methods of improving ADR reporting among healthcare professionals, Uganda, 2013**

| Method | Freqency | Percentage |
|---|---|---|
| Sensitize, train and give continuous medical education to healthcare professionals | 666 | 42.0 |
| Make forms available e.g. on wards in patient hospital files | 262 | 16.5 |
| Sensitize the public through media, posters and counsel patients about ADRs | 159 | 10.5 |
| Create liaison office to coordinate ADR reportg in each health facility | 74 | 4.6 |
| Incentivize reporting/Motivate health workers/Provide Financial support | 65 | 4.1 |
| Provide toll-free telephone line or Online ADR reporting system | 58 | 3.6 |
| Increase and strengthen onsite support/supervision | 38 | 2.4 |
| Compulsory ADR reporting | 23 | 1.4 |
| Give feedback to ADR reporters | 21 | 1.3 |
| Increase awareness of existence of the National Pharmacovigilance Centre | 21 | 1.3 |
| Other | 202 | 13.0 |
| **TOTAL** | **1,589** | **100%** |

For peer review only

## Appendix

| **Assessment of Adverse Drug Reaction Reporting among Healthcare Professionals in Uganda** | |
|---|---|
| **Investigator:** ____________ | **District:** ____________ |
| An **Adverse Drug Reaction** (ADR) is any response to a drug which is harmful and unintended, and which occurs at doses normally used by patients. | |
| **HEALTH FACILITY CHARACTERISTICS** | |
| **1. Type of health facility** (*Tick one only*)<br>[1] Public<br>[2] Private Not-for-Profit<br>[3] Private For-Profit | **2. Level of health facility** (*Tick one only*)<br>[1] National Referral [5] Health Centre III<br>[2] Regional Referral [6] Health Centre II<br>[3] District Hospital [7] Private Hospital<br>[4] Health Centre IV [8] Other………….. |
| **SOCIO-DEMOGRAPHIC CHARACTERISTICS OF PARTICIPANT** | |
| **3. Gender**<br>[1] Male<br>[2] Female | **4. How old are you (in complete years)?** ……….. |
| **PROFESSIONAL CHARACTERISTICS OF PARTICIPANT** | |
| **5. In which sector(s) do you practice**? (*Tick all that apply*)<br>[1] Public health facility<br>[2] Private Not-for-Profit health facility<br>[3] Private For-Profit health facility | **6. In which department are you?** (*Tick one only*)<br>[1] Medicine<br>[2] Surgery<br>[3] Paediatrics<br>[4] Obstetrics & Gynaecology<br>[5] Dentistry<br>[6] Pharmacy<br>[7] Other (Specify)……….. |
| **7. What is the approximate number of patients you see per day**? …………… | **8. For how long have you been working in this health facility?** ....... Months (If less than 1 year)<br>….. Completed Years |
| **9. What is your highest academic qualification?** (*Tick one only*)<br>[1] Certificate<br>[2] Diploma<br>[3] First Degree<br>[4] Masters Degree<br>[5] PhD | **10. For how long have you been practicing since you qualified with your highest academic training?**<br>…… Months (If less than 1 year)<br>…… Completed Years |

For peer review only

| | |
|---|---|
| **11. Do you teach medical students?**<br><br>[1] Yes [2] No (If no, **go to 13**) | **12. If yes, duration of practice in a teaching hospital**<br>…… Months (If less than 1 year)<br>…… Completed Years |
| **13. Are you actively involved in medical research?**<br><br>[1] Yes [2] No | **14. Professional Cadre** (*Tick one only*)<br>[1] Doctor (**go to 15**)<br>[2] Pharmacist (**go to 22**)<br>[3] Nurse (**go to 19**)<br>[4] Clinical officer (**go to 23**)<br>[5] Pharmacy Technician (**go to 22**)<br>[6] Other (Specify)……………………… |
| **15. Position/Level of Doctor** (*Tick one only*)<br>[1] Senior Consultant<br>[2] Consultant<br>[3] Medical Officer Special Grade<br>[4] Medical Officer<br>[5] Senior House Officer<br>[6] Intern Doctor<br>[7] Other (specify)………… | **16. For how long have you been prescribing?**<br>…… Months (If less than 1 year)<br>…… Completed Years |
| **17. What is the approximate number of prescriptions you write per day?**............. | **18. Have you given verbal prescriptions/orders to the attending nurse in the past 12 months?**<br>[1] Yes [2] No<br>(***Skip to 23***) |
| **19. Which of the following cadre category describes your qualification?** (*Tick one only*)<br>[1] Enrolled Midwife<br>[2] Enrolled Nurse<br>[3] Enrolled Mental Health Nurse<br>[4] Enrolled Comprehensive Nurse<br>[5] Registered Midwife<br>[6] Registered Nurse<br>[7] Registered Nurse/Midwife<br>[8] Registered Mental Health Nurse<br>[9] Registered Comprehensive Nurse<br>[10] Other (specify)…………………… | **20. In some health facilities, nurses usually write out (transcribe) drug prescriptions from patients' medical records to medication charts. Are you required to transcribe prescriptions in your health facility?**<br>[1] Yes [2] No |
| **21. In practice, do you regularly transcribe prescriptions?**<br>[1] Yes [2] No<br>(***Skip to 23***) | **22. If pharmacist or pharmacy technician, area of practice** (*Tick all that apply*)<br>[1] Hospital [3] Academia<br>[2] Industry [4] Community/Private |

For peer review only

**SUSPECTED ADVERSE DRUG REACTION (ADR) REPORTING PROGRAM**

**23. Have you received any complaint of adverse drug reactions (ADRs) from patients in the last 4 weeks?**

[1] Yes [2] No (If no, **go to 25**)

**24. If yes, how many complaints of ADRs have you received in the last 4 weeks?** …….

**25. Have you suspected an ADR in the last 4 weeks?**

[1] Yes [2] No (If no, **go to 28**)

**26. If yes, how many ADRs have you suspected in the last 4 weeks?** ...........

**27. Briefly describe the most recent suspected ADR you encountered providing information on patient age, drug involved & route of administration, outcome of ADR & its severity (mild, moderate, severe); e.t.c.**

…………………………………………………………………………………………………………
…………………………………………………………………………………………………………
…………………………………………………………………………………………………………
…………………………………………………………………………………………………………
…………………………………………………………………………………………………………

**28. Have you ever encountered a fatal ADR that might have led to a patient's death?**

[1] Yes [2] No

**29. Have you reported any suspected ADR in the last 12 months?**

[1] Yes [2] No (If No, **go to 35**)

**30. If yes, please indicate the period within which you reported the most recent suspected ADR**

| [1] | [2] | [3] | [4] | [5] |
|---|---|---|---|---|
| **4 weeks** | **5-8 weeks** | **9-12 weeks** | **4-6 mo** | **7-12 mo** |

**31. To which authorities did you report the most recent of these ADRs?**
(*Tick all that apply*)

[1] National Drug Authority (NDA)

[2] AIDS Treatment Information Centre (ATIC)

[3] Drug Manufacturer

[4] Medical Superintendent

[5] District Director of Health Services (DDHS)

[6] Institutional Review Board (IRB)

[7] Other (specify)………………..............

**32. What motivated you to report the suspected ADR?**

…………………………………….
…………………………………….
…………………………………….

**33. Did you get any feedback about the ADR report(s) you submitted?**

[1] Yes [2] No

**34. Have you reported an ADR to the National Drug Authority in the past 12 months?**

[1] Yes [2] No

**35. Have you wanted to report an ADR in the past 12 months but did not have the ADR report form?**

[1] Yes [2] No

**36. Have you had an ADR suspicion in the past 12 months but did not fill the ADR report form even when you had it?**

**37. Did you ever fill the ADR report form but failed to send it for any reason?**

[1] Yes [2] No (If no, **go to 39**)

For peer review only

| | |
|---|---|
| [1] Yes [2] No | |
| **38. If yes, what was the reason(s) that you did not send the form on the most recent occasion?**<br>………………………………….....<br>………………………………….....<br>………………………………….....<br>…………………………………..... | **39. Which of the following health workers are qualified to report adverse drug reactions?** (Tick all that apply)<br>[1] Medical doctors [4] Pharmacists<br>[2] Dentists [5] Clinical Officers<br>[3] Nurses |
| **40. Pharmacovigilance relates to a reporting system for adverse effects of medicines. Have you ever heard about Pharmacovigilance?**<br>[1] Yes [2] No (If no, **go to 42**) | **41. If yes, please state the source(s) of your information** (Tick all that apply)<br>[1] Books/Journals<br>[2] Internet/e-communication<br>[3] Trainings/Seminars/courses attended<br>[4] Television<br>[5] Outdoor adverts<br>[6] Professional colleague<br>[7] Others (Specify)………………………… |
| **42. Are you aware of the existence of a National Pharmacovigilance Centre (NPC) in Uganda?**<br>[1] Yes [2] No (If no, **go to 44**) | **43. If yes, do you know where the NPC office is located?**<br>[1] Yes [2] No |
| **44. Have you ever seen the ADR form used for reporting ADRs to the NPC?**<br>[1] Yes [2] No (If no, **go to 48**) | **45. If yes, have you ever filled out the NPC ADR form?**<br>[1] Yes [2] No (If no, **go to 47**) |
| **46. Was the information on the NPC ADR form clear to you about what to report?**<br>[1] Yes [2] No | **47. Have you ever filled out any ADR form different from that of the NPC?**<br>[1] Yes [2] No |
| **48. Have you ever submitted an ADR report to the NPC?**<br>[1] Yes [2] No | **49. Do you know where to obtain the NPC ADR forms in this health facility?**<br>[1] Yes [2] No |
| **50. Do you know to whom to report ADRs in your health facility?**<br>[1] Yes [2] No (If no, **go to 52**) | **51. If yes, please specify in your health facility to whom you would report an ADR if you had to?**<br>…………………………………...<br>…………………………………... |
| **52. An ADR reporting system should;** (*Tick all that apply*)<br>[1] be compulsory<br>[2] be voluntary<br>[3] provide financial incentives to the reporter | **53. Have you ever been trained on how to report ADRs with the ADR form?**<br>[1] Yes [2] No |

For peer review only

| | |
|---|---|
| [4] hide the identity of the prescriber<br>[5] hide the identity of the reporter<br>[6] hide the identity of the patient | |
| **54. Please suggest possible ways of improving ADR reporting**<br>........................................................................<br>........................................................................ | |

**Instructions**

In the left column are questions that will be the subject of your evaluation and in the right column is a gradual scale where you should mark with **X** the place along the scale where, according to your opinion, represents your degree of agreement with the text comment. The **extreme left** side indicates **total disagreement** while the **extreme right** indicates **total agreement**. Agreement increases as you move across from left to right
Please indicate whether you agree or disagree with the following statements
(1 = Strongly disagree; 2 = Slightly disagree; 3 = Neutral; 4 = Slightly agree; 5 = Strongly agree)

Strongly Disagree ←→ Strongly Agree

| | Statement | 1 | 2 | 3 | 4 | 5 |
|---|---|---|---|---|---|---|
| 55 | Serious ADRs are well documented by the time a drug is marketed | | | | | |
| 56 | It is nearly impossible to determine whether a drug is responsible for a particular adverse reaction | | | | | |
| 57 | I would only report an ADR if I was sure that it was related to the use of a particular drug | | | | | |
| 58 | The one case of an ADR that an individual health worker might see makes no significant contribution to medical knowledge | | | | | |
| 59 | I read articles about adverse drug reactions with interest | | | | | |
| 60 | I have a professional obligation to report ADRs | | | | | |
| 61 | Reporting ADRs puts my career at risk | | | | | |
| 62 | It is only necessary to report serious or unexpected ADRs | | | | | |
| 63 | I do not have time to complete an ADR report form | | | | | |
| 64 | I do not have the time to actively look for ADRs while at work | | | | | |
| 65 | I do not know how information reported in ADR form is used | | | | | |
| 66 | I talk with pharmaceutical companies about possible ADRs with their drugs | | | | | |
| 67 | I think that the best way to report ADRs is by publishing in medical literature | | | | | |
| 68 | I should be financially reimbursed for providing the ADR service | | | | | |
| 69 | I would be more likely to report ADRs if there were an easier method | | | | | |

For peer review only

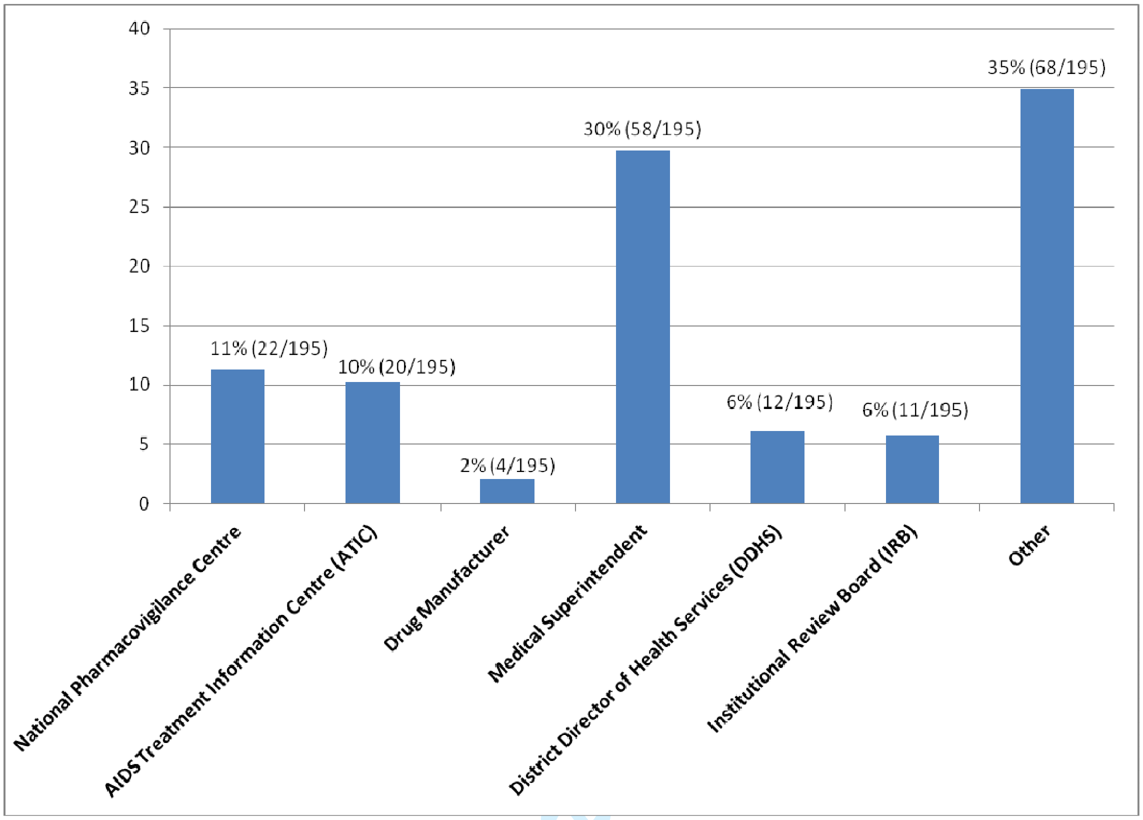


Fig 1: Authorities to whom 178 healthcare professionals most frequently reported a recent suspected ADR (% responses)

For peer review only

BMJ Open

# Recognition and reporting of suspected adverse drug reactions by surveyed healthcare professionals in Uganda: key determinants

| | |
|---|---|
| Journal: | *BMJ Open* |
| Manuscript ID: | bmjopen-2014-005869.R1 |
| Article Type: | Research |
| Date Submitted by the Author: | 22-Jul-2014 |
| Complete List of Authors: | Kiguba, Ronlad; Makerere University College of Health Sciences, Pharmacology and Therapeutics<br>Karamagi, Charles; Makerere University College of Health Sciences, Clinical Epidemiology Unit, Department of Medicine<br>Waako, Paul; Makerere University College of Health Sciences, Pharmacology and Therapeutics<br>Ndagije, Helen; National Drug Authority, National Pharmacovigilance Centre<br>Bird, Sheila; MRC Biostatistics Unit, |
| <b>Primary Subject Heading</b>: | Health services research |
| Secondary Subject Heading: | Epidemiology, Health policy, Medical education and training, Pharmacology and therapeutics |
| Keywords: | pharmacoviligance, adverse drug reactions, reporting-rates, Uganda, healthcare professionals |
| | |

SCHOLARONE™
Manuscripts

# Recognition and reporting of suspected adverse drug reactions by surveyed healthcare professionals in Uganda: key determinants

*Ronald Kiguba[1], Charles Karamagi[2], Paul Waako[1], Helen B. Ndagije[3] and Sheila M. Bird[4]*

[1]Department of Pharmacology and Therapeutics, Makerere University College of Health Sciences, Kampala, Uganda

[2]Clinical Epidemiology Unit, Department of Medicine, Makerere University College of Health Sciences, Kampala, Uganda

[3]National Pharmacovigilance Centre, National Drug Authority, Kampala, Uganda

[4]Medical Research Council Biostatistics Unit, Cambridge, United Kingdom

* Corresponding author

Email addresses:

RK: kiguba@gmail.com

CK: ckaramagi2000@yahoo.com

PW: paulwaako@yahoo.com

HBN: hndagije@gmail.com

SMB: sheila.bird@mrc-bsu.cam.ac.uk

For peer review only

# ABSTRACT

**Objective**

To assess extent and determinants of past-month recognition of suspected adverse drug reactions (ADR) and past-year ADR reporting among healthcare professionals (HCPs) in Uganda.

**Setting**

Geographically diverse health facilities (public, private for-profit, private not-for-profit).

**Participants**

Of 2,000 questionnaires distributed, 1,345 were completed: return-rate of 67%.

**Primary and secondary outcome measures**

Percent HCPs who suspected ADR in past-month; reported ADR in past-year.

**Results**

Nurses were the majority (58%, 776/1,340). Only half the respondents had heard about pharmacovigilance: 39% of nurses (295/763; 95% CI: 35% to 42%), 70% otherwise (383/547; 95% CI: 66% to 74%). One fifth (268/1,289 or 21%; 95% CI: 19% to 23%) had suspected an ADR in the previous 4 weeks, 111 of them nurses; 15% (190/1,296) had reported a suspected ADR in the past-year, 103 of them nurses.

Past-month ADR suspicion was more likely by non-nurses (odds ratio (OR) = 1.7, 95% CI: 1.16 – 2.40) and with medical research involvement (OR = 1.5, 95% CI: 1.05 – 2.15) but past-month receipt of patient ADR-complaint predominated (OR = 19, 95% CI: 14-28).

Past-year ADR reporting was higher by hospital staff (OR = 1.9, 95% CI: 1.18-3.10), especially in medicine (OR = 2.3, 95% CI: 1.08-4.73); but lower from private for-profit health facilities (OR = 0.5, 95% CI: 0.28-0.77) and by older staff (OR = 0.6, 95% CI: 0.43-0.91); more likely by HCPs who had ever encountered a fatal ADR (OR = 2.9, 95% CI: 1.94-4.25), knew to whom to report (OR = 1.7, 95% CI: 1.18-2.46), or suggested how to improve ADR

For peer review only

reporting (OR = 1.6, 95% CI: 1.04-2.49). Two attitudinal factors were important: diffidence and lethargy.

**Conclusions**

One in five HCPs suspected an ADR in the past-month and one in seven reported ADR in the previous-year. Empowering patients could strengthen ADR detection and reporting in Africa.

## Strengths

Over 1,300 healthcare professionals surveyed in diverse health facilities in Uganda
Attitudes to pharmacovigilance elicited
Demographic and professional determinants ascertained of past-month ADR suspicion and past-year ADR reporting.

## Limitations

Purposely-selected survey locations and non-random sampling of healthcare professionals
Under-representation of nurses.

For peer review only

## Background

Adverse drug reactions (ADRs) are significant causes of patient morbidity and mortality[1] and are known to raise overall healthcare costs[2-5]. The World Health Organization (WHO) defines pharmacovigilance (PV) as "the science and activities relating to the detection, assessment, understanding and prevention of adverse effects or any other medicine-related problem"[6]. Spontaneous and voluntary reporting of suspected ADRs generates signals about rare, delayed and elsewhere have estimated that only 6-10% of all ADRs unexpected drug reactions that are undetected in the initial phases of drug development[7] but under-reporting is a major limitation[8]. Studies conducted are reported[9-11]. This low rate of ADR reporting undermines efforts to identify and estimate the magnitude of drug risks, confirmation of actionable issues, and possible regulatory action[12].

Widespread use of electronic medical record databases has enhanced patient safety through automation of signal detections for ADRs, thereby improving healthcare service delivery[13]. In Africa, the establishment and use of such databases is still rare [14] and ADR reporting is largely done manually. Strengthening of PV systems in sub-Saharan African (SSA) countries has received support from global health initiatives, but reporting is often disease specific (e.g. malaria, vaccines, HIV/AIDS) because of restricted funding streams rather than strengthening countrywide reporting systems[15]. As a result, PV systems in SSA remain weak[16]. In Uganda, 556 spontaneous reports were submitted to the National Pharmacovigilance Centre (NPC) in the initial five years of 2005-2009. Of these, 315 (57%) were related to medicines with 10 or more spontaneous ADR reports and were dominated by antiretroviral drugs (51%, 160/315), antimalarials (27%, 85/315), and antibiotics (22%, 70/315)[17]. The dominance of ADR reports related to these groups of medicines accords with the burden of disease in SSA[18].

The WHO's Uppsala Monitoring Centre (UMS) maintains web-based ADR reporting software (VigiFlow) for use by National Pharmacovigilance Centres[19]. Although receipt of 200 or more ADR reports per million population per year is desirable[20], most SSA countries

For peer review only

submit fewer than 20 ADR reports per million population in 2010 compared to more than 100 reports per million in other low- and middle-income countries[21].

Uganda established a National Pharmacovigilance Centre (NPC) in 2005 and has been a member of the WHO program for International Drug Monitoring since 2007. In 2010, there was a training-of-trainers session for 30 national pharmacovigilance trainers. By 2011, 14 regional PV centres were established[21], PV-training sessions for core teams of healthcare professionals (HCPs) were conducted in each of these centres, and ADR reporting forms distributed[22]. At least one support supervision visit per centre is conducted annually. Despite these efforts, reporting rate in Uganda (population: 36 million) is still low at 6 ADR reports per million population per year, based on 1,348 ADR reports in 2007-2012 [180, 75, 229[23], 140, 183, 413 in 2012 (when Targeted Spontaneous Reporting (TSR) was launched); and 128 in January-June 2013 (Nassali Huldah & Helen Ndagije, personal communication, 15 Jan 2014)]. Moreover, significant missing information in four-fifths of ADR reports compromises analysis[17].

Of 46 SSA countries whose PV systems were assessed to determine their capacity to ensure drug safety, Uganda was identified as one of four with active PV systems that could, in principle, detect, evaluate, and address medicine safety issues[24]. Indeed, Ugandan surgical series[25] on, and subsequent media coverage of, gluteal fibrosis and post injection paralysis among children injected with quinine[26, 27] triggered investigation by the Ugandan NPC which, in 2010, mediated change of Uganda's recommended quinine injection site from the gluteus muscle to the thigh[28].

Personal and professional characteristics associated with increased ADR reporting by HCPs include older age, male gender, lower workload, higher number of prescriptions issued per day, type of education received, specific PV training, and involvement in teaching and research[8, 29, 30]. Inhibitory factors include: unavailability of ADR forms, bureaucratic method of ADR reporting, and uncertainty over which professional cadre is mandated to report ADRs[31].

For peer review only

In 1996, Inman et al[32] described eight 'deadly sins' to explain why HCPs underreport ADRs: i) attitudes related to professional activities (financial incentives, fear of litigation, and ambition to publish personal case series), ii) ADR–related knowledge and attitudes (complacency, diffidence, indifference, and ignorance), and iii) excuses made by HCPs (lethargy). Insecurity is an attitudinal factor that was not proposed by Inman but has been reported elsewhere[33].

In Africa, there is a paucity of empirical data on PV awareness[34-38]. Hence we sought to determine the level of PV awareness by HCPs, and the extent and determinants of past-month ADR recognition and of past-year ADR reporting in Uganda.

## Methods

### Study design and sampling procedure

From 25 May 2012 through 28 February 2013, we conducted a survey across Uganda in purposively selected, geographically diverse public and private health facilities. Public institutions included the National Referral Hospital-Mulago, and six Regional Referral Hospitals each selected to represent a major region of the country. In addition, we included District Hospitals and Health Centres (HCs) at levels II to IV in the catchment area where a Regional Referral Hospital was selected. For logistical reasons, we selected a convenience sample of private for-profit and private not-for-profit health facilities (which included drug shops) in the respective districts where public institutions were assessed. Permission to conduct the research was sought from the administrators of the selected institutions.

Any HCP involved in prescribing, transcribing, dispensing medication orders, and administration of drugs to a patient was eligible for inclusion. Written informed consent was obtained from HCPs prior to their recruitment. The self-completed questionnaires did not contain identifying information on individual HCPs. The survey team used serial numbers to track distributed questionnaires. Five research assistants, all final year medical students at Mulago National Referral Hospital, were initially recruited and trained on the concepts of pharmacovigilance, informed consent, response rate and on the survey questionnaire which

For peer review only

they self-completed. Completion of questionnaire by research assistants was primarily to familiarize them with it and to gauge time to completion (22, 25, 27, 31 and 31; mean of 27 minutes) but served also as a brief pre-test. A similar model of data collection by pre-trained investigators was employed in the upcountry sites.

Given the challenge of accessing staff lists in the selected health facilities (and especially so in private-for-profit settings), random sampling of eligible HCPs was not practicable. Instead, in each health facility, the pre-trained investigators approached HCPs of all ranks and invited them to complete a pretested questionnaire, of which 2,200 were printed and 2,000 distributed. Invitations might be declined if HCPs were particularly busy or, despite willingness, a delay of several days or weeks might ensue before the self-completion questionnaire was returned. In practice, neither the refusal-rate by approached HCPs nor the 'did not return rate', by professional cadre, for distributed questionnaires was reliably documented.

In Uganda, there were reckoned to be 46,566 HCPs in 2009[39], who would have been survey-eligible had they worked at the survey-locations. Doctors and dentists (3,459) represented an estimated 7% of the nationally eligible staff but were 20% of the achieved sample; 762 pharmacists and pharmacy technicians 1.6% of nationally eligible staff but 6% of the achieved sample; and 37,625 nurses, midwives and nursing assistants an estimated 81% of the nationally eligible staff but 59% of the achieved sample.

**Data collection and management**

The survey questionnaire, see **Appendix**, elicited demographic and professional information, description of the most recent suspected ADR, and attitudes to, as well as knowledge and use of, the suspected ADR reporting system. The questionnaire for HCPs included 15 attitudinal statements on ADR reporting which were scored from 1 (total disagreement) to 5 (total agreement). All data were entered into a databank using EpiData 3.1.

Prior to its administration, the questionnaire was elaborated between members of the research team who have diverse expertise in pharmacy, pharmacovigilance, and questionnaire design.

For peer review only

Completion-time was tested by research assistants. Thereafter, an integrated pilot study was conducted on 125 healthcare professionals. The subsequent revisions were sufficiently minor that results of the pre-test were included in the final analysis.

**Statistical analysis**

Responses are summarized as frequencies and percentages. Different potential determinants for the past-month recognition or past-year reporting of suspected ADRs were screened using $\chi^2$-tests for categorical variables. Logistic regression was then used to assess the relationship of demographic and professional factors severally to: i) recognition of suspected ADRs in the past 4 weeks; and for those in post for at least one year, ii) having reported at least one suspected ADR in the past 12 months. Attitudinal factors were also incorporated in ii). Missing data were accounted for using multiple imputations under the missing at random assumption[40] on the one hand or, as here, the missing-assigned approach on the other, where missing data were meaningfully assigned to an existing category. Results are expressed as odds ratios (ORs) with 95% confidence intervals. Statistical analyses were carried out using Stata 12.0[41].

**Ethical clearance**

Ethical approval was obtained from the School of Medicine Research and Ethics Committee, Makerere University College of Health Sciences, and the Uganda National Council for Science and Technology.

## Results

**Study population**

Of 2,000 questionnaires distributed, 1,345 were completed, a return-rate of 67%. Mean age of respondent HCPs was 32.4 years (SD = 8.9). Nurses were the majority (776/1,340 or 58%), see **Table 1**.

**Awareness of pharmacovigilance**

For peer review only

Half the respondents (678/1,310 or 52%; 95% CI: 49% to 55%) had ever heard about pharmacovigilance: two-fifths of nurses (295/763 or 39%; 95% CI: 35% to 42%) but 70% of others (383/547; 95% CI: 66% to 74%). Thirty percent of HCPs (412/1,317; 95% CI: 29% to 34%) were aware of the existence of Uganda's NPC but only 3% (37/1,312; 95% CI: 2% to 4%) of HCPs had *ever* submitted an ADR report to the NPC.

**Suspected ADR reporting in the previous 12 months**

Only 15% of HCPs (190/1,296; 95% CI: 13% to 17%) had reported a suspected ADR in the previous 12 months, of whom 15% (27/175) claimed to have made their report to NPC so that our respondents' past-year ADR reporting rate to NPC was an estimated 1 in 50 (2%). Only 41% (11/27; 95% CI: 22% to 59%) past-year reporters to NPC had found the NPC-form clear on what to report.

When HCPs were asked about when, in the past 12 months, they had reported their most recent suspected ADR, 79/178 (44%) said within the past month, 28 (16%) in the months 2+3 prior, and 71 (40%) in months 4-12, a distribution indicative either of a multiplicity of reports per ADR-reporter or biased recall.

**ADR recognition**

Twenty one per cent (268/1,289: 95% CI, 19% to 23%) of respondents had suspected an ADR in the previous one month, 76% of whom (195/257: 95% CI, 71% to 81%) had received patient ADR-complaints in the past month. Of HCPs who had suspected an ADR in the past month, 35% (92/262: 95% CI, 29% to 41%) had reported an ADR in the past 12 months. Among HCPs who had not suspected an ADR in the previous month, 12% (121/1,000: 95% CI, 10% to 14%) had nonetheless received patient ADR-complaints in the past month.

In the previous 4 weeks, see **Table 2**, 26% (340/1,302) of HCPs had received 1,190 patient ADR-complaints [mean of 3.5 complaints (sd 9.5) per complaint-receiving HCP] which equates to 0.9 ADR-complaints (95% CI: 0.65 to 1.18) per HCP per month. Also, 21%

For peer review only

(268/1,289) of HCPs had suspected 670 ADRs [mean of 2.5 suspected ADRs (sd 2.6) per suspecting HCP] which equates to 0.5 suspected ADRs (95% CI: 0.45 to 0.59) per HCP per month, implying an ADR suspicion rate of 0.57 (0.52/0.91) per patient ADR-complaint per HCP per month (95% CI: 0.42 to 0.80).

Among the 15% (190/1,296) who were ADR-reporters in the previous 12 months, 44% (79/178) claimed to have submitted their most recent report in the past 4 weeks. If so, there could be at least 84 suspected ADR reports submitted by 1,296 HCPs in the past 4 weeks (or 0.065 ADR-reports in past 4 weeks per HCP) when 0.5 ADRs were suspected in the past 4 weeks per HCP. This translates into a 13% ADR-report rate per suspected ADR.

**Medication classes and fatalities in survey-described suspected ADRs**

The most frequently mentioned medication classes associated with 182 survey-described ADRs in the past 4-weeks which cited one or more drugs (216 drug citations) were antibiotics (38%, 83/216), antiretroviral agents (23%, 49/216), antimalarials (15%, 33/216, 15 of which implicated quinine), analgesics (9%, 19/216), and others (15%, 32/216).

Two suspected ADRs were described by HCPs which involved child fatalities in association with quinine: a 5-year-old girl had been given intravenous quinine and died soon after arrival at a private-not-for-profit hospital in Eastern Uganda; and a 2-year old boy had reacted to quinine and died despite the doctor in a public hospital in Eastern Uganda having administered an antidote. Full details of HCPs' described suspected ADRs will be reported separately.

**Feedback to ADR reporters**

Reporters of ADRs to AIDS Treatment Information Centre (ATIC) received the highest feedback (60%, 12/20), followed by those who reported to the Medical Superintendent or Institutional Review Board (39%: 23/58 + 4/11). Feedback from Uganda's NPC was infrequent (23%: 5/22). Reporters of ADRs to drug manufacturers (4) or District Directors of Health Services (12) received zero feedback.

For peer review only

**Reasons for ADR reporting**

The commonest reason that respondents vouched for ADR reporting was that the patient had developed a serious ADR (30%, 48/159 reasons) followed by patient safety (18%, 29/159), and patient ADR-complaint (8%, 13/159). The next three reasons each had nine citations: institutional mandate to report ADRs, prevention of similar ADRs, and as a means of obtaining advice.

**Attitudes to ADR reporting**

Only 14% (186/1,301: 95% CI, 12% to 16%) of respondents indicated that reporting ADRs put their career at risk, see **Table 3**, while 36% (466/1,304: 95% CI, 33% to 38%) thought that it is only necessary to report serious or unexpected ADRs. Most respondents agreed that they have a professional obligation to report ADRs (76%, 1,000/1,311: 95% CI, 74% to 79%) and 68% (896/1,319: 95% CI, 65% to 70%) stated that they would report ADRs if there were an easier method. Forty five per cent (596/1,312: 95% CI, 43% to 48%) stated that they do not know how information reported in the ADR form is used, 64% (833/1,309: 95% CI, 61% to 66%) felt that they would report an ADR only if they were sure it was related to use of a particular drug, and 27% (349/1,305: 95% CI, 24% to 29%) felt that they should be financially reimbursed for providing the ADR reporting service.

**Factors associated with ADR suspicion in the past month**

Suspicion of ADR in the past 4-weeks was more likely by non-nurses (odds ratio (OR) = 1.7, 95% CI: 1.16 – 2.40) and with involvement in medical research (OR = 1.5, 95% CI: 1.05 – 2.15) but the clearly dominant factor was that the HCP had received patient ADR-complaint(s) in the past 4-weeks (OR = 19, 95% CI: 14-28). There was some evidence that ADR suspicion was less likely by staff in surgical wards, see **Table 4**.

Logistic regression analysis among the 973 respondents who did not receive a patient ADR complaint did not identify any additional significant cofactors associated with ADR suspicion.

**Personal, professional and attitudinal factors associated with having made an ADR report in the past 12 months**



For peer review only

Demographic and professional factors associated with a lower likelihood to report ADRs in the past 12 months were: private for-profit health facility (vs. public; OR = 0.5, 95% CI: 0.28 – 0.77) and HCP aged over 30 years (OR = 0.6, 95% CI: 0.43 – 0.91); while those associated with being more likely to report ADRs included: medical department (vs. surgery; OR = 2.3, 95% CI: 1.08 – 4.73), having ever encountered a fatal ADR (OR = 2.9, 95% CI: 1.94 – 4.25), knowing to whom to report ADRs (OR = 1.7, 95% CI: 1.18 – 2.46), and HCPs who had suggested ways of improved ADR reporting (OR = 1.6, 95% CI: 1.04 – 2.49), see **Table 5.** Only two attitudinal factors were additionally relevant: diffidence ('the belief that reporting an ADR would only be done if there was certainty that it was related to the use of a particular drug'; OR = 0.6, 95% CI: 0.41-0.89) and lethargy ('I do not know how information reported in ADR form is used'), see **Table 6**.

**Suggestions for improved ADR reporting**

The most frequently cited suggestion was to sensitize, train and provide ongoing medical education on ADRs to HCPs (42%, 667/1,589 suggestions) followed by making ADR forms available (17%, 262/1,589), sensitizing the public and counselling patients about ADRs (11%, 166/1,589), creating a coordinating office in each health facility (5%, 73/1,589), providing financial incentives to reporters (4%, 65/1,589), and making available telephone or online ADR reporting systems (4%, 57/1,589), see **Table 7**.

## Discussion

A low proportion of HCPs reported having submitted an adverse drug reaction (ADR) report in the previous 12 months (15%) and the level of awareness of PV was also low, similar to observations made elsewhere[34, 42, 43]. Healthcare professionals from different cadres may recognize suspected ADRs but fail to take the responsibility to report[44]. Barely one in eight (13%) of suspected ADRs in the past month was reported by the HCPs in that same period, yet around three-fifths of patient ADR-complaints in the past month were adjudged by HCPs to be suspected ADRs. Integration of pharmacovigilance into pre-service training curricula

For peer review only

and emphasizing its importance in promoting patient safety in healthcare delivery is a first step[45, 46] upon which other PV initiatives can build.

To raise the number of submitted ADR reports, Uganda has proposed mandatory reporting of ADRs by industry and HCPs[22]. However, questions have been raised about the effectiveness of compulsory reporting by HCPs[47] and the NPC needs to improve its feedback to ADR reporters since our respondents ranked it much lower than ATIC. Moreover, HCPs in our study reported ADRs to a greater extent than in nationally-reported statistics: 2% of HCPs (27/1281: 95% CI, 1.3% to 2.9%) had reported any suspected ADR to the NPC in the previous year compared with the NPC's annual average national ADR reporting rate for Uganda from 2007 to mid-2013 of 0.44% [based on 1,348 reports in 6.5 years from 46,566 clinical staff countrywide: 95% CI, 0.38% to 0.51%] or 0.90% in the highest report-year of 2012 [413 reports in 2012: 95% CI, 0.80% to 0.97%]. Thus, HCPs in our study seemed at least twice as likely to have submitted suspected ADRs to the NPC in the previous year when compared with the national ADR reporting rates by Uganda's HCPs.

One limitation to our estimates is that more than one HCP may have described (and reported) the same suspected ADR since our ability to discriminate between suspected ADRs was compromised by variation in the quality of ADR descriptions, a limitation that NPC also contends with.

Consistent with ADR reports from the NPC[17], we identified antibiotics, antiretroviral agents, and antimalarials as the three most frequently cited medication classes in survey-described ADRs. Therefore, health initiatives already focusing on the PV of these medications, if replicated for other classes, present opportunities to strengthen overall PV systems in these settings[17]. As a PV exemplar in Uganda, the NPC and AIDS Control Program introduced TSR in 2011 to monitor tenofovir for renal toxicity and to detect suspected ADRs related to antiretroviral therapy use in the Prevention of Mother to Child Transmission of HIV and in the Early Infants Diagnosis program[48]. Results from TSR are yet to be disseminated, however.

For peer review only

Around three-fifths of patients' ADR-complaints to HCPs in the past month translated into ADR suspicion. Patient ADR-complaint was dominant among explanatory factors for HCPs' ADR-suspicion in the past month and so we suggest that empowering patients to support HCPs may improve the detection and reporting of suspected ADRs. Moreover, other countries have instituted systems that promote spontaneous direct patient reporting of suspected ADRs thus permitting patients to participate in PV activities that teach them to handle their medicines better and improves their communication with HCPs[49, 50].

Improvement of the ADR reporting form for Uganda seems necessary. Therefore, our research team designed a form that is relevant to the inpatient setting and captures additional information required for causality assessment of suspected medicines. This form will be tested in a follow-up study on inpatients.

Other suggestions to improve ADR reporting by respondents included; increased visibility of the NPC and giving useful feedback to ADR reporters, introducing telephone and online reporting systems, increasing onsite support supervision, making ADR forms more available, providing training and continued medical education of HCPs as suggested elsewhere[51], and sensitizing the public to ADRs. The absence of a national PV policy, however, coupled with the lack of proper coordination between the NPC and numerous health programmes and sentinel sites may undermine efforts to strengthen the countrywide PV system[17]. For example, in Uganda's teaching hospitals, could some clinical grand rounds address PV and suspected serious ADRs?

Although previous studies suggested a positive relationship between older age and ADR reporting[52, 53], we found that older HCPs (≥30 years) were less likely than their younger counterparts to have reported suspected ADRs in the past 12 months. These contrasting results might be attributed to idiosyncratic differences between HCPs and healthcare systems in Europe and Africa such that younger staff, as in our study, may have had more PV training. There is, as yet, limited published literature from other African settings. Our respondents were, on average, 10 years younger when compared with studies conducted in Europe[29]. We

suggest that older HCPs in Uganda be targeted in future strategies on improved ADR reporting.

In contrast to other studies[53], training on how to report ADRs was not significantly associated with increased ADR reporting. Given the cross-sectional study design we used, it was not possible to establish whether PV training preceded ADR reporting, or vice versa, and therefore we were unable to assess their temporal relationship. That notwithstanding, Gonzalez-Gonzalez *et al* have suggested that multifaceted interventions, as opposed to single educational programmes, increase to a greater extent HCPs' PV awareness and motivate them to report ADRs[8].

A low level of PV awareness may lead to underreporting of ADRs[54]. In our study, knowing to whom to report was an important factor for ADR reporting in the final logistic regression. We also observed that the proportion (31%: 95% CI, 29% to 34%) of respondents aware of the existence of Uganda's NPC is lower than reported for Nigeria [52% (51/99): 95% CI, 42% to 61%][34]. Much higher proportions of PV awareness have been reported in Europe[29] and Asia[55, 56] where there are higher ADR reporting rates per million of population[57] and more government involvement in national PV programs[34].

Healthcare professionals who had ever encountered a fatal ADR were twice as likely to report an ADR as HCPs who had not. Correspondingly, development of a serious or fatal ADR was the most frequently cited reason for ADR reporting. We also found that HCPs who suggested possible ways of improving the ADR reporting system were more likely to have reported an ADR in the previous 12 months[58].

Healthcare professionals who agreed with the statement 'I would only report an ADR if I was sure that it was related to the use of a particular drug' (diffidence) were less likely to report suspected ADRs. Apart from diffidence and lethargy/indifference ('I do not know how information reported in the ADR form is used'), none of the other Inman factors was associated with ADR reporting[8, 32, 59]. Diffidence and lethargy can be targeted in educational interventions to promote ADR reporting and by improved feedback to ADR-reporters.

Although provision of financial incentives to reporters was the fifth most frequently cited suggestion to improve ADR reporting, it was not statistically significant in the logistic regression for the odds on ADR reporting and these findings are consistent with those in the developed world[60].

In private for-profit health facilities, HCPs were less likely to have reported ADRs in the previous 12 months than their counterparts in the public sector. In addition, HCPs in hospitals (public and private) were twice as likely as those from other health facilities (HCs II & III, community pharmacies, drug shops) to have reported suspected ADRs in the previous 12 months. Whereas few PV scale-up activities in Africa have given priority to the private sector[16, 22], more public-private collaboration could strengthen PV systems in our SSA setting[61].

Our study had several limitations. First, we used self-report as the main method of inquiry and this may have introduced recall bias. Second, we may have experienced social desirability bias as HCPs may not have given frank responses for fear of being embarrassed if they were not reporting ADRs. However, as we used self-administered questionnaires without respondents' names, the potential for this bias was reduced. Third, the cross-sectional design that we used could not establish temporal relationships between ADR reporting in past year and some explanatory factors. Fourth, there was over-representation of doctors and pharmacists/pharmacy technicians versus nurses. Finally, several respondents may have referred to the same suspected ADR but this did not have a significant bearing since our main focus was assessment of individual ADR reporting behaviour rather than on individual ADRs. Our study has, however, generated key insights on determinants in Uganda for HCPs' ADR suspicion and reporting.

## Conclusions

One in five HCPs had suspected an ADR in the past 4 weeks while one in seven had reported an ADR in the previous 12 months. Empowering patients to support HCPs in suspected ADR

For peer review only

detection and reporting is essential to strengthening PV systems in Africa. HCPs who ever encountered fatal ADRs are keener reporters and can consequently help others to avoid the experience that made them better reporters. HCPs ought to know that they don't have to be certain about causality to report suspected ADRs. Poor access to suspected ADR forms and lack of feedback on reports are constraints that can be rectified. [4,279 words]

For peer review only

**FOOTNOTES**

## Authors' contributions

RK conceived of the study and drafted the manuscript and, in conjunction with SMB, participated in its design, implementation, statistical analysis and the drawing of inferences. CK, PW and HBN participated in study design and in the process of manuscript writing. All authors approved the final manuscript.

## Competing Interests

SMB holds GSK shares. The authors declare that they otherwise have no competing interests.

## Funding

This work was supported by Training Health Researchers into Vocational Excellence (THRiVE) in East Africa, grant number 087540, funded by the Wellcome Trust; grant number 5R24TW008886 supported by OGAC, NIH and HRSA; and an African Doctoral Dissertation Research Fellowship award offered by the African Population and Health Research Centre

(APHRC) in partnership with the International Development Research Centre (IDRC). In UK, SMB is funded by Medical Research Council programme number MC_U105260794.

## Role of funders

The work here reported is solely the responsibility of the authors and does not necessarily represent the official views of the supporting offices. The funders had no role in the decisions on what and where to publish.

## Data sharing

Categorical data are available from the lead author, Ronald Kiguba, by email request to kiguba@gmail.com.

## Acknowledgements

The authors wish to thank all the HCPs who agreed to participate in this study.

We also thank Huldah Nassali at the National Pharmacovigilance Centre for providing technical support to this project.

Finally, RK thanks Noeline Nakasujja at Makerere University College of Health Sciences, and Yukari C. Manabe at Johns Hopkins University for supporting his development of the first three drafts of the manuscript.

For peer review only

References


1. Lazarou J, Pomeranz BH, Corey PN. Incidence of adverse drug reactions in hospitalized patients: a meta-analysis of prospective studies. *JAMA* 1998; **279**(15): 1200-5.
2. Ramesh M, Pandit J, Parthasarathi G. Adverse drug reactions in a south Indian hospital--their severity and cost involved. *Pharmacoepidemiol Drug Saf* 2003; **12**(8): 687-92.
3. Classen DC, Pestotnik SL, Evans RS, et al. Adverse drug events in hospitalized patients. Excess length of stay, extra costs, and attributable mortality. *JAMA* 1997; **277**(4): 301-6.
4. Bates DW, Spell N, Cullen DJ, et al. The costs of adverse drug events in hospitalized patients. Adverse Drug Events Prevention Study Group. *JAMA* 1997; **277**(4): 307-11.
5. Gautier S, Bachelet H, Bordet R, Caron J. The cost of adverse drug reactions. *Expert Opin Pharmacother* 2003; **4**(3): 319-26.
6. World Health Organization. The Safety of Medicines in Public Health Programmes: Pharmacovigilance an Essential Tool. 2006. http://www.who.int/medicines/areas/quality_safety/safety_efficacy/Pharmacovigilance_B.pdf (accessed 31 May 2014).
7. Lexchin J. Is there still a role for spontaneous reporting of adverse drug reactions? *CMAJ : Canadian Medical Association journal = journal de l'Association medicale canadienne* 2006; **174**(2): 191-2.
8. Lopez-Gonzalez E, Herdeiro MT, et al. Determinants of under-reporting of adverse drug reactions: a systematic review. *Drug Saf* 2009; **32**(1): 19-31.
9. Hazell L, Shakir SA. Under-reporting of adverse drug reactions : a systematic review. *Drug Saf* 2006; **29**(5): 385-96.
10. Smith CC, Bennett PM, Pearce HM, et al. Adverse drug reactions in a hospital general medical unit meriting notification to the Committee on Safety of Medicines. *Br J Clin Pharmacol* 1996; **42**(4): 423-9.
11. Pirmohamed M, Breckenridge AM, Kitteringham NR, et al. Adverse drug reactions. *BMJ* 1998; **316**(7140): 1295-8.
12. Gavaza P, Brown CM, Lawson KA, et al. Influence of attitudes on pharmacists' intention to report serious adverse drug events to the Food and Drug Administration. *Br J Clin Pharmacol* 2011; **72**(1): 143-52.
13. Miguel A, Azevedo LF, Lopes F, et al. Methodologies for the detection of adverse drug reactions: comparison of hospital databases, chart review and spontaneous reporting. *Pharmacoepidemiol Drug Saf* 2013; **22**(1): 98-102.
14. Alj L, Touzani MDW, Benkirane R, Edwards IR, Soulaymani R. Detecting medication errors in pharmacovigilance database: Capacities and limits. *Int J Risk Saf Med* 2007; **19**: 187-94.
15. Strengthening Pharmaceutical Systems (SPS). Supporting Pharmacovigilance in Developing Countries: The Systems Perspective
Arlington, VA: Submitted to the U.S. Agency for International Development by the SPS Program, 2009.
16. Isah AO, Pal SN, Olsson S, et al. Specific features of medicines safety and pharmacovigilance in Africa. *Therapeutic Advances in Drug Safety* 2012; **3**(1): 25-34.
17. National Pharmacovigilance Centre. Working together with the Public Health Programmes: a regulator's perspective for addressing the minimum requirements for Pharmacovigilance. 2010. http://www.who.int/medicines/areas/quality_safety/regulation_legislation/icdra/WG-2_2Dec.pdf (accessed 21 June 2013).
18. Institute for Health Metrics and Evaluation. The Global Burden of Disease: Generating Evidence, Guiding Policy-Sub-Saharan Africa Regional Edition. 2013.


For peer review only

http://www.healthdata.org/policy-report/global-burden-disease-generating-evidence-guiding-policy%E2%80%94sub-saharan-africa-regional (accessed 31 May 2014).
19. Uppsala Monitoring Centre (UMC). VigiFlow ™ The complete Individual Case Safety Report (ICSR) management system from the UMC. http://www.who-umc.org/graphics/27705.pdf (accessed 31 May 2014).
20. Uppsala Monitoring Centre (UMC). Pharmacovigilance - Reporting Trends. 2013. http://who-umc.org/DynPage.aspx?id=108476&mn1=7347&mn2=7252&mn3=7322&mn4=7558 (accessed 31 May 2014).
21. Strengthening Pharmaceutical Systems (SPS) Program. Safety of Medicines in sub-Saharan Africa: Assessment of Pharmacovigilance Systems and their Performance. 2011. http://apps.who.int/medicinedocs/documents/s19152en/s19152en.pdf2014).
22. Ouma C, Abwao E. Africa Pharmacovigilance Meeting 2012: Technical Report. Submitted to the US Agency for International Development by the Systems for Improved Access to Pharmaceuticals and Services Program, 2012.
23. National Pharmacovigilance Centre. The Pharmacovigilance News. 2010. http://www.nda.or.ug/docs/bulletin%20issue%20No.1.pdf (accessed 1 June 2014).
24. Appiah B. Africa struggles to improve drug safety. *CMAJ : Canadian Medical Association journal = journal de l'Association medicale canadienne* 2012; **184**(10): E533-4.
25. Ekure J. Gluteal Fibrosis. A report of 28 cases from Kumi Hospital, Uganda. *East and Central African Journal of Surgery* 2006; **12**(1): 144-7.
26. Ekure J. Post-injection paralysis: The new polio in Uganda. 9 December 2007. http://www.newvision.co.ug/D/9/34/601055.
27. Chinnock P. Concern in Uganda over children injured by poorly administered malaria treatment 2009. http://www.tropika.net/svc/news/20090604/Chinnock-20090604-News-Quinine-Uganda (accessed 13 May 2014).
28. National Pharmacovigilance Centre. What is current practice of Pharmacovigilance in Uganda? 2012. http://africapv2012.files.wordpress.com/2012/04/day-2_5_country-presentations-nras_h-ngadije-uganda-compatibility-mode.pdf (accessed 11 December 2013).
29. Bateman DN, Sanders GL, Rawlins MD. Attitudes to adverse drug reaction reporting in the Northern Region. *Br J Clin Pharmacol* 1992; **34**(5): 421-6.
30. Figueiras A, Tato F, Fontainas J, et al. Influence of physicians' attitudes on reporting adverse drug events: a case-control study. *Med Care* 1999; **37**(8): 809-14.
31. Rawlins MD. Pharmacovigilance: paradise lost, regained or postponed? The William Withering Lecture 1994. *J R Coll Physicians Lond* 1995; **29**(1): 41-9.
32. Inman WH. Attitudes to adverse drug reaction reporting. *Br J Clin Pharmacol* 1996; **41**(5): 434-5.
33. Herdeiro MT, Figueiras A, Polonia J, et al. Physicians' attitudes and adverse drug reaction reporting : a case-control study in Portugal. *Drug Saf* 2005; **28**(9): 825-33.
34. Oshikoya KA, Awobusuyi JO. Perceptions of doctors to adverse drug reaction reporting in a teaching hospital in Lagos, Nigeria. *BMC Clin Pharmacol* 2009; **9**: 14.
35. Robins AH, Weir M, Biersteker EM. Attitudes to adverse drug reactions and their reporting among medical practitioners. *South African medical journal = Suid-Afrikaanse tydskrif vir geneeskunde* 1987; **72**(2): 131-4.
36. Ball D, Tisocki T. Adverse drug reaction reporting by general medical practitioners and retail pharmacists in Harare--a pilot study. *Cent Afr J Med* 1998; **44**(8): 190-5.
37. Okezie EO, Olufunmilayo F. Adverse drug reactions reporting by physicians in Ibadan, Nigeria. *Pharmacoepidemiol Drug Saf* 2008; **17**(5): 517-22.
38. Jaquet A, Djima MM, Coffie P, et al. Pharmacovigilance for antiretroviral drugs in Africa: lessons from a study in Abidjan, Cote d'Ivoire. *Pharmacoepidemiol Drug Saf* 2011; **20**(12): 1303-10.
39. Africa Health Workforce Observatory. Human Resources for Health Country Profile - Uganda. 2009. p. 30.

For peer review only

40. Sterne JA, White IR, Carlin JB, et al. Multiple imputation for missing data in epidemiological and clinical research: potential and pitfalls. *BMJ* 2009; **338**: b2393.
41. StataCorp. Stata Statistical Software: Release 12. College Station, TX: StataCorp LP; 2011.
42. Khan SA, Goyal C, Chandel N, et al. Knowledge, attitudes, and practice of doctors to adverse drug reaction reporting in a teaching hospital in India: An observational study. *Journal of natural science, biology, and medicine* 2013; **4**(1): 191-6.
43. Pimpalkhute SA, Jaiswal KM, Sontakke SD, et al. Evaluation of awareness about pharmacovigilance and adverse drug reaction monitoring in resident doctors of a tertiary care teaching hospital. *Indian journal of medical sciences* 2012; **66**(3-4): 55-61.
44. Bemt van den PMLA, Egberts ACG. Drug-related problems: definitions and classifications. *EJHP* 2007; (13): 62-4.
45. Gallagher TH, Waterman AD, Ebers AG, et al. Patients' and physicians' attitudes regarding the disclosure of medical errors. *JAMA* 2003; **289**(8): 1001-7.
46. Cox AR, Marriott JF, Wilson KA, et al. Adverse drug reaction teaching in UK undergraduate medical and pharmacy programmes. *J Clin Pharm Ther* 2004; **29**(1): 31-5.
47. Cross C. Drug reporting may be mandatory for doctors. *CMAJ : Canadian Medical Association journal = journal de l'Association medicale canadienne* 2013; **185**(10): 866.
48. Uppsala Monitoring Centre. Uppsala Reports 59. 2012.
49. Andrew Herxheimer, Rose Crombag, Teresa Leonardo Alves. Direct Patient Reporting of Adverse Drug Reactions. May 2010.
50. Masso Guijarro P, Aranaz Andres JM, et al. Adverse events in hospitals: the patient's point of view. *Quality & safety in health care* 2010; **19**(2): 144-7.
51. Bukirwa H, Nayiga S, Lubanga R, et al. Pharmacovigilance of antimalarial treatment in Uganda: community perceptions and suggestions for reporting adverse events. *Trop Med Int Health* 2008; **13**(9): 1143-52.
52. Tubert-Bitter P, Haramburu F, et al. Spontaneous reporting of adverse drug reactions: who reports and what? *Pharmacoepidemiol Drug Saf* 1998; **7**(5): 323-9.
53. Irujo M, Beitia G, Bes-Rastrollo M, et al. Factors that influence under-reporting of suspected adverse drug reactions among community pharmacists in a Spanish region. *Drug Saf* 2007; **30**(11): 1073-82.
54. Ohaju-Obodo JO, Iribhogbe OI. Extent of pharmacovigilance among resident doctors in Edo and Lagos states of Nigeria. *Pharmacoepidemiol Drug Saf* 2010; **19**(2): 191-5.
55. Li Q, Zhang SM, Chen HT, et al. Awareness and attitudes of healthcare professionals in Wuhan, China to the reporting of adverse drug reactions. *Chin Med J (Engl)* 2004; **117**(6): 856-61.
56. Chatterjee S, Lyle N, Ghosh S. A survey of the knowledge, attitude and practice of adverse drug reaction reporting by clinicians in Eastern India. *Drug Saf* 2006; **29**(7): 641-2.
57. Aagaard L, Strandell J, Melskens L, et al. Global patterns of adverse drug reactions over a decade: analyses of spontaneous reports to VigiBase. *Drug Saf* 2012; **35**(12): 1171-82.
58. Passier A, ten Napel M, van Grootheest K, et al. Reporting of adverse drug reactions by general practitioners: a questionnaire-based study in the Netherlands. *Drug Saf* 2009; **32**(10): 851-8.
59. Herdeiro MT, Figueiras A, Polonia J, et al. Influence of pharmacists' attitudes on adverse drug reaction reporting : a case-control study in Portugal. *Drug Saf* 2006; **29**(4): 331-40.
60. Herdeiro MT, Polonia J, Gestal-Otero JJ, et al. Factors that influence spontaneous reporting of adverse drug reactions: a model centralized in the medical professional. *J Eval Clin Pract* 2004; **10**(4): 483-9.
61. World Health Organization (WHO) - MMV joint technical consultation on active pharmacovigilance monitoring with a special focus on affordable medicine facility for malaria (AMFm). Pharmacovigilance Guidance for Countries Participating in AMGm Phase 1. 2009. http://apps.who.int/medicinedocs/documents/s18427en/s18427en.pdf (accessed 1 June 2014).

For peer review only

For peer review only

**Table 1: Demographic and professional characteristics of healthcare professionals, Uganda, 2013**

| | | |
|---|---|---|
| **Total number of participants** | | **1,345** |
| **Age, n = 1,253** | | |
| Mean years (SD); median, inter-quartile range | 32.4 ( 8.9); 30, 26-36 | |
| **Gender, n = 1,345** | | |
| Male | | 541 (40.2) |
| Female | | 804 (59.8) |
| **Number of Patients seen per day, n = 1,226** | | |
| Mean number (SD); median, inter-quartile range | 41.0 (46.3); 30, 15-50 | |
| **Professional Cadre, n = 1,340** | | |
| Nurse | | 792 (59.1) |
| Doctor | | 275 (20.5) |
| Pharmacist & Pharmacy Technician | | 84 ( 6.3) |
| Other | | 44 ( 3.3) |
| **Type of Health Facility, n = 1,345** | | |
| Public | | 568 (42.2) |
| Private Not-For-Profit | | 280 (20.8) |
| Private-For-Profit | | 497 (37.0) |
| **Highest Academic Qualification, n = 1,345** | | |
| Certificate | | 471 (35.0) |
| Diploma | | 501 (37.3) |
| First Degree | | 294 (21.9) |
| Masters Degree or PhD | | 79 ( 5.9) |
| **Ever received ADR training, n = 1,225** | | |
| Yes | | 180 (14.7) |
| No | | 1045 (85.3) |
| **Received Patient ADR Complaint in past 4 weeks, n = 1,302** | | |
| Yes | | 340 (26.1) |
| No | | 962 (73.9) |

For peer review only

**Table 2: Patient ADR complaints and healthcare professional ADR suspicion in past 4 weeks, Uganda, 2013**

| **Patient ADR-Complaints/Healthcare Professional ADR suspicion** | | | | | |
|---|---|---|---|---|---|
| **Patient ADR-Complaints in past 4 weeks** | | | | | |
| Cadre | No of HCPs | Who received complaints | Mean (SD) ADR-Complaints | ADR-complaints received | ADR-complaints per HCP |
| Overall | 1,302 | 340 (26%) | 3.5 ( 9.5) | 1,190 | 0.91 |
| **Nurses** | **760** | **155 (20%)** | **3.9 (11.4)** | **604** | **0.80** |
| **Non-nurses** | **542** | **185 (34%)** | **3.2 ( 7.7)** | **592** | **1.09** |
| Doctors | 270 | 97 (36%) | 3.3 (10.2) | 320 | 1.19 |
| Pharm/Ptech | 81 | 34 (42%) | 3.9 ( 4.0) | 132 | 1.64 |
| Other | 191 | 54 (28%) | 2.5 ( 2.1) | 135 | 0.71 |
| **Healthcare Professionals' ADR suspicion in past 4 weeks** | | | | | |
| Cadre | No of HCPs | Who suspected ADRs | Mean (SD) suspected ADRs | ADR Suspicions by HCPs | ADR-suspicion per HCP |
| Overall | 1,289 | 268 (21%) | 2.5 (2.6) | 670 | 0.52 |
| **Nurses** | **756** | **111 (15%)** | **2.6 (2.6)** | **288** | **0.38** |
| **Non-nurses** | **533** | **157 (29%)** | **2.5 (2.6)** | **393** | **0.74** |
| Doctors | 267 | 88 (33%) | 2.3 (2.5) | 202 | 0.76 |
| Pharm/Ptech | 80 | 23 (29%) | 2.9 (3.2) | 66 | 0.83 |
| Other | 186 | 46 (25%) | 2.5 (2.5) | 114 | 0.61 |

For peer review only

**Table 3: Healthcare professionals' responses to 15 attitudinal statements on ADR reporting, Uganda, 2013**

| Statement | Agree | Neutral | Disagree |
|---|---|---|---|
| Serious ADRs are well documented by the time a drug is marketed | 820 (61.7) | 166 (12.5) | 343 (25.8) |
| It is nearly impossible to determine whether a drug is responsible for a particular adverse reaction | 527 (39.8) | 189 (14.3) | 607 (45.9) |
| I would only report an ADR if I was sure that it was related to the use of a particular drug | 833 (63.6) | 138 (10.6) | 338 (25.8) |
| The one case of an ADR that an individual health worker might see makes no significant contribution to medical knowledge | 210 (16.2) | 122 (9.4) | 966 (74.4) |
| I read articles about adverse drug reactions with interest | 824 (63.3) | 180 (13.8) | 298 (22.9) |
| I have a professional obligation to report ADRs | 1000 (76.3) | 143 (10.9) | 168 (12.8) |
| Reporting ADRs puts my career at risk | 186 (14.3) | 126 (9.7) | 989 (76.0) |
| It is only necessary to report serious or unexpected ADRs | 466 (35.7) | 129 (9.9) | 709 (54.4) |
| I do not have time to complete an ADR report form | 143 (10.9) | 208 (15.8) | 963 (73.3) |
| I do not have the time to actively look for ADRs while at work | 195 (14.8) | 152 (11.6) | 968 (73.6) |
| I do not know how information reported in ADR form is used | 596 (45.4) | 194 (14.8) | 522 (39.8) |
| I talk with pharmaceutical companies about possible ADRs with their drugs | 290 (22.2) | 202 (15.5) | 813 (62.3) |
| I think the best way to report ADRs is by publishing in medical literature | 701 (53.4) | 238 (18.1) | 374 (28.5) |
| I should be financially reimbursed for providing the ADR service | 349 (26.7) | 199 (15.3) | 757 (58.0) |
| I would be more likely to report ADRs if there were an easier method | 896 (67.9) | 169 (12.8) | 254 (19.3) |

For peer review only

**Table 4: Personal and professional factors associated with ADR suspicion in the past 4 weeks among 1,289 healthcare professionals, Uganda, 2013**

| Factor | ADR Suspicion | | Crude Analysis | | | Adjusted Analysis | | |
|---|---|---|---|---|---|---|---|---|
| | Yes (%) | No (%) | OR | 95%CI | P-value | OR | 95%CI | P-value |
| Level of Health Facility | | | | | | | | |
| Other | 77 (16.1) | 413 (83.9) | 1.0 | | | 1.0 | | |
| Hospital | 191 (23.5) | 621 (76.5) | 1.6 | 1.19-2.14 | 0.002 | 1.3 | 0.81-2.06 | 0.286 |
| Type of Health Facility | | | | | | | | |
| Public | 129 (23.2) | 426 (76.8) | 1.0 | | | 1.0 | | |
| Private Not-For-Profit | 55 (20.5) | 213 (79.5) | 0.9 | 0.60-1.22 | 0.380 | 0.8 | 0.51-1.27 | 0.353 |
| Private For-Profit | 84 (18.0) | 382 (82.0) | 0.7 | 0.53-0.99 | 0.041 | 0.8 | 0.49-1.30 | 0.362 |
| Region of the country | | | | | | | | |
| Central | 148 (25.3) | 437 (74.7) | 1.0 | | | 1.0 | | |
| Eastern | 62 (15.1) | 348 (84.9) | 0.5 | 0.38-0.73 | <0.001 | 0.6 | 0.37-0.94 | 0.025 |
| Other | 58 (19.7) | 236 (80.3) | 0.7 | 0.52-1.02 | 0.066 | 0.8 | 0.50-1.22 | 0.270 |
| **Professional Cadre** | | | | | | | | |
| Nurse | 111 (14.7) | 645 (85.3) | 1.0 | | | 1.0 | | |
| Non-nurse | 157 (29.5) | 376 (70.5) | 2.4 | 1.84-3.19 | <0.001 | 1.7 | 1.16-2.40 | 0.005 |
| Age | | | | | | | | |
| Less than 30 years | 119 (20.8) | 452 (79.2) | 1.0 | | | 1.0 | | |
| Aged 30 years or older | 149 (20.8) | 569 (70.3) | 1.0 | 0.76-1.30 | 0.969 | 0.9 | 0.65-1.31 | 0.647 |
| Patient Load | | | | | | | | |
| Greater than 30/day | 128 (22.2) | 449 (77.8) | 1.0 | | | 1.0 | | |
| At most 30/day | 140 (19.7) | 572 (80.3) | 0.9 | 0.66-1.12 | 0.268 | 1.2 | 0.85-1.75 | 0.272 |
| Department | | | | | | | | |
| Surgery | 13 (13/1) | 86 (86.9) | 1.0 | | | 1.0 | | |
| Medicine | 150 (23.7) | 482 (76.3) | 2.1 | 1.12-3.79 | 0.021 | 2.1 | 0.99-4.38 | 0.054 |
| Paediatrics, Obs&Gyn | 40 (20.2) | 158 (79.8) | 1.7 | 0.85-3.30 | 0.136 | 2.0 | 0.90-4.57 | 0.090 |
| Other | 65 (18.1) | 295 (81.9) | 1.5 | 0.77-2.77 | 0.250 | 1.4 | 0.66-3.18 | 0.358 |
| **Involved in medical research** | | | | | | | | |
| No | 160 (17.6) | 749 (82.3) | 1.0 | | | 1.0 | | |
| Yes | 108 (38.6) | 272 (61.4) | 1.9 | 1.40-2.46 | <0.001 | 1.5 | 1.05-2.15 | 0.026 |
| Ever encountered Fatal ADR | | | | | | | | |
| No | 197 (19.0) | 842 (81.0) | 1.0 | | | 1.0 | | |
| Yes | 71 (28.4) | 179 (71.6) | 1.7 | 1.24-2.32 | 0.001 | 1.1 | 0.71-1.64 | 0.732 |
| Knowing to whom to report | | | | | | | | |
| No | 129 (20.2) | 511 (79.8) | 1.0 | | | 1.0 | | |
| Yes | 139 (21.4) | 510 (78.6) | 1.1 | 0.82-1.41 | 0.577 | 1.2 | 0.86-1.74 | 0.254 |
| Suggestions for improved ADR reporting | | | | | | | | |
| No | 54 (17.0) | 264 (83.0) | 1.0 | | | 1.0 | | |
| Yes | 214 (22.0) | 757 (78.0) | 1.4 | 0.99-1.92 | 0.054 | 0.9 | 0.60-1.37 | 0.628 |

For peer review only

| | | | | | | | | |
|---|---|---|---|---|---|---|---|---|
| **Received patient ADR complaint in past 4 weeks** | | | | | | | | |
| No | 73 ( 7.5) | 900 (92.5) | 1.0 | | | 1.0 | | |
| Yes | 195 (61.7) | 121 (38.3) | 19.9 | 14.3-27.6 | <0.001 | 19.0 | 13.5-27.1 | <0.001 |

For peer review only

**Table 5: Personal and professional factors associated with ADR reporting in the past 12 months among 1,164 healthcare professionals who had been in post for at least 1 year, Uganda, 2013**

| Factor | ADR Reporter | | Crude Analysis | | | Adjusted Analysis | | |
|---|---|---|---|---|---|---|---|---|
| | Yes (%) | No (%) | OR | 95%CI | P-value | OR | 95%CI | P-value |
| Level of Health Facility | | | | | | | | |
| Other | 36 (8.0) | 413 (92.0) | 1.0 | | | 1.0 | | |
| Hospital | 128 (17.9) | 587 (82.1) | 2.5 | 1.69-3.70 | <0.001 | 1.9 | 1.18-3.10 | 0.008 |
| Type of Health Facility | | | | | | | | |
| Public | 91 (18.5) | 402 (81.5) | 1.0 | | | 1.0 | | |
| Private Not-For-Profit | 40 (16.8) | 198 (83.2) | 0.9 | 0.59-1.34 | 0.585 | 0.8 | 0.50-1.23 | 0.286 |
| Private For-Profit | 33 (7.6) | 400 (92.4) | 0.4 | 0.24-0.56 | <0.001 | 0.5 | 0.28-0.77 | 0.003 |
| Region of the country | | | | | | | | |
| Central | 82 (15.9) | 433 (84.1) | 1.0 | | | 1.0 | | |
| Eastern | 36 (9.7) | 334 (90.3) | 0.6 | 0.38-0.86 | 0.008 | 0.7 | 0.43-1.13 | 0.140 |
| Other | 46 (16.5) | 233 (83.5) | 1.0 | 0.70-1.55 | 0.836 | 1.2 | 0.75-1.84 | 0.471 |
| Professional Cadre | | | | | | | | |
| Nurse | 93 (13.5) | 597 (86.5) | 1.0 | | | 1.0 | | |
| Non-nurse | 71 (15.0) | 403 (85.0) | 1.1 | 0.81-1.58 | 0.470 | 0.8 | 0.55-1.18 | 0.264 |
| Age | | | | | | | | |
| Less than 30 years | 70 (15.0) | 396 (85.0) | 1.0 | | | 1.0 | | |
| Aged 30 years or older | 94 (13.5) | 604 (86.5) | 0.9 | 0.63-1.23 | 0.455 | 0.6 | 0.43-0.91 | 0.014 |
| Patient Load | | | | | | | | |
| Greater than 30/day | 84 (16.1) | 439 (83.9) | 1.0 | | | 1.0 | | |
| At most 30/day | 80 (12.5) | 561 (87.5) | 0.7 | 0.54-1.04 | 0.081 | 0.9 | 0.61-1.27 | 0.510 |
| Department | | | | | | | | |
| Surgery | 10 (11.5) | 77 (88.5) | 1.0 | | | 1.0 | | |
| Medicine | 95 (16.3) | 488 (83.7) | 1.5 | 0.75-3.00 | 0.253 | 2.3 | 1.08-4.73 | 0.030 |
| Paediatrics, Obs&Gyn | 18 (10.5) | 153 (89.5) | 0.9 | 0.40-2.06 | 0.065 | 0.8 | 0.36-1.95 | 0.675 |
| Other | 41 (12.7) | 282 (87.3) | 1.1 | 0.54-2.34 | 0.147 | 1.6 | 0.73-3.50 | 0.243 |
| Involved in medical research | | | | | | | | |
| No | 103 (12.6) | 716 (87.4) | 1.0 | | | 1.0 | | |
| Yes | 61 (17.7) | 284 (82.3) | 1.5 | 1.06-2.11 | 0.023 | 1.3 | 0.88-1.87 | 0.191 |
| Ever encountered Fatal ADR | | | | | | | | |
| No | 98 (10.7) | 820 (89.3) | 1.0 | | | 1.0 | | |
| Yes | 62 (27.1) | 167 (72.9) | 3.0 | 2.12-4.33 | <0.001 | 2.9 | 1.94-4.25 | <0.001 |
| Knowing to whom to report | | | | | | | | |
| No | 62 (11.0) | 504 (89.1) | 1.0 | | | 1.0 | | |
| Yes | 102 (17.1) | 496 (82.9) | 1.7 | 1.19-2.35 | 0.003 | 1.7 | 1.18-2.46 | 0.005 |
| Suggestions for improved ADR reporting | | | | | | | | |
| No | 32 (10.6) | 270 (89.4) | 1.0 | | | 1.0 | | |
| Yes | 132 (15.3) | 730 (84.7) | 1.5 | 1.01-2.30 | 0.044 | 1.6 | 1.04-2.49 | 0.032 |

For peer review only

**Table 6: Attitudinal factors associated with ADR reporting in past 12 months among 1,114 healthcare professionals who responded to attitudinal questions, Uganda, 2013**

| Factor | Reported an ADR in the past 12 months | | Crude Analysis | | | Adjusted Analysis* | | |
|---|---|---|---|---|---|---|---|---|
| | Yes (%) | No (%) | OR | 95% CI | P-value | OR | 95% CI | P-value |
| I do not know how information reported in ADR form is used | | | | | | | | |
| Agree | 64 (12.5) | 447 (87.5) | 0.7 | 0.47-0.97 | 0.031 | 0.7 | 0.46-1.00 | 0.052 |
| Neutral | 17 (10.6) | 143 (89.4) | 0.6 | 0.32-0.98 | 0.041 | 0.5 | 0.27-0.94 | 0.030 |
| Disagree | 81 (17.5) | 383 (82.5) | 1.0 | | | 1.0 | | |
| I would only report an ADR if I was sure that it was related to the use of a particular drug | | | | | | | | |
| Agree | 86 (12.2) | 620 (87.8) | 0.6 | 0.39-0.81 | 0.002 | 0.6 | 0.41-0.89 | 0.011 |
| Neutral | 12 (9.9) | 109 (90.1) | 0.4 | 0.23-0.87 | 0.015 | 0.6 | 0.29-1.17 | 0.128 |
| Disagree | 60 (19.7) | 244 (80.3) | 1.0 | | | 1.0 | | |

*Adjusted for personal and professional characteristics: level of health facility, type of health facility, region, non-nurse as professional cadre, age, patient load, department, involvement in medical research, ever encountered a fatal ADR, knowing to whom to report ADRs, and suggesting ways to improve ADR reporting

For peer review only

**Table 7: Suggested methods of improving ADR reporting among healthcare professionals, Uganda, 2013**

| Method | Freqency | Percentage |
|---|---|---|
| Sensitize, train and give continuous medical education to healthcare professionals | 666 | 42.0 |
| Make forms available e.g. on wards in patient hospital files | 262 | 16.5 |
| Sensitize the public through media, posters and counsel patients about ADRs | 159 | 10.5 |
| Create liaison office to coordinate ADR reportg in each health facility | 74 | 4.6 |
| Incentivize reporting/Motivate health workers/Provide Financial support | 65 | 4.1 |
| Provide toll-free telephone line or Online ADR reporting system | 58 | 3.6 |
| Increase and strengthen onsite support/supervision | 38 | 2.4 |
| Compulsory ADR reporting | 23 | 1.4 |
| Give feedback to ADR reporters | 21 | 1.3 |
| Increase awareness of existence of the National Pharmacovigilance Centre | 21 | 1.3 |
| Other | 202 | 13.0 |
| **TOTAL** | **1,589** | **100%** |

For peer review only



For peer review only - http://bmjopen.bmj.com/site/about/guidelines.xhtml

# Recognition and reporting of suspected adverse drug reactions by surveyed healthcare professionals in Uganda: key determinants

*Ronald Kiguba[1]*, Charles Karamagi[2], Paul Waako[1], Helen B. Ndagije[3] and Sheila M. Bird[4]*

[1]Department of Pharmacology and Therapeutics, Makerere University College of Health Sciences, Kampala, Uganda

[2]Clinical Epidemiology Unit, Department of Medicine, Makerere University College of Health Sciences, Kampala, Uganda

[3]National Pharmacovigilance Centre, National Drug Authority, Kampala, Uganda

[4]Medical Research Council Biostatistics Unit, Cambridge, United Kingdom

* Corresponding author

Email addresses:

RK: kiguba@gmail.com

CK: ckaramagi2000@yahoo.com

PW: paulwaako@~~chs.mak.ac.ug~~yahoo.com

HBN: hndagije@gmail.com

SMB: sheila.bird@mrc-bsu.cam.ac.uk

Field Code Changed



For peer review only

For peer review only - http://bmjopen.bmj.com/site/about/guidelines.xhtml

# ABSTRACT

**Objective**

To assess extent and determinants of past-month recognition of suspected adverse drug reactions (ADR) and past-year ADR reporting among healthcare professionals (HCPs) in Uganda.

**Setting**

Geographically diverse health facilities (public, private for-profit, private not-for-profit).

**Participants**

Of 2,000 questionnaires distributed, 1,345 were completed:, a return-rate of 67%.1,345 HCPs, two-thirds of those to whom questionnaire was distributed.

**Primary and secondary outcome measures**

Percent HCPs who suspected ADR in the past-month; reported ADR in the past-year.

**Results**

Nurses were the majority (58%, 776/1,340). Only half the respondents had heard about pharmacovigilance: 39% of nurses (295/763; 95% CI: 35% to 42%), 70% otherwise (383/547; 95% CI: 66% to 74%). One fifth (268/1,289 or 21%; 95% CI: 19% to 23%) had suspected an ADR in the previous 4 weeks, 111 of them nurses; 15% (190/1,296) had reported a suspected ADR in the past-year, 103 of them nurses.

Past-month ADR suspicion was more likely by non-nurses (odds ratio (OR) = 1.7, 95% CI: 1.16 – 2.40) and with medical research involvement (OR = 1.5, 95% CI: 1.05 – 2.15) but past-month receipt of patient ADR-complaint predominated (OR = 19, 95% CI: 14-28).




For peer review only - http://bmjopen.bmj.com/site/about/guidelines.xhtml

Past-year ADR reporting was higher by hospital staff (OR = 1.9, 95% CI: 1.18-3.10), especially in medicine (OR = 2.3, 95% CI: 1.08-4.73); but lower from private for-profit health facilities (OR = 0.5, 95% CI: 0.28-0.77) and by older staff (OR = 0.6, 95% CI: 0.43-0.91); more likely by HCPs who had ever encountered a fatal ADR (OR = 2.9, 95% CI: 1.94-4.25), knew to whom to report (OR = 1.7, 95% CI: 1.18-2.46), or suggested how to improve ADR reporting (OR = 1.6, 95% CI: 1.04-2.49). Two attitudinal factors were important: diffidence and lethargy.

**Conclusions**

One in five HCPs suspected an ADR in the past-month and one in seven reported an ADR in the previous-year. Empowering patients could strengthen ADR detection and reporting in Africa.

## Strengths

- Over 1,300 healthcare professionals surveyed in diverse health facilities in Uganda
- Return-rate of self-completion questionnaire was two-thirds
- Attitudes to pharmacovigilance elicited
- Demographic and professional determinants ascertained of past-month ADR suspicion and past-year ADR reporting.

Formatted: Highlight

## Limitations

- Purposely-selected survey locations and non-random sampling of healthcare professionals
- Non-random sampling of healthcare professionals
- Self-report as the main method of inquiry
- Temporal relationship between past-year ADR reporting and some explanatory factors (patient-ADR-complaint in the past-month) could not be determined
- Under-representation of nurses



For peer review only - http://bmjopen.bmj.com/site/about/guidelines.xhtml

- Several respondents may have referred to the same suspected ADR but this did not have a significant bearing since our main focus is assessment of individual ADR reporting behaviour rather than individual ADRs.

For peer review only



For peer review only - http://bmjopen.bmj.com/site/about/guidelines.xhtml

# Recognition and reporting of suspected adverse drug reactions by surveyed healthcare professionals in Uganda: key determinants

*Ronald Kiguba[1], Charles Karamagi[2], Paul Waako[1], Helen B. Ndagije[3] and Sheila M. Bird[4]*

[1]Department of Pharmacology and Therapeutics, Makerere University College of Health Sciences, Kampala, Uganda

[2]Clinical Epidemiology Unit, Department of Medicine, Makerere University College of Health Sciences, Kampala, Uganda

[3]National Pharmacovigilance Centre, National Drug Authority, Kampala, Uganda

[4]Medical Research Council Biostatistics Unit, Cambridge, United Kingdom

## Background

Adverse drug reactions (ADRs) are significant causes of patient morbidity and mortality[1] and are known to raise overall healthcare costs[2-5]. The World Health Organization (WHO) defines pharmacovigilance (PV) as “the science and activities relating to the detection, assessment, understanding and prevention of adverse effects or any other medicine-related problem”[6]. Spontaneous and voluntary reporting of suspected ADRs generates signals about rare, delayed and unexpected drug reactions that are undetected in the initial phases of drug development[7] but under-reporting is a major limitation[8]. Studies conducted elsewhere have estimated that only 6-10% of all ADRs are reported[9-11]. This low rate of ADR reporting undermines efforts to identify and estimate the magnitude of drug risks, confirmation of actionable issues, and possible regulatory action[12].



For peer review only

For peer review only - http://bmjopen.bmj.com/site/about/guidelines.xhtml

Widespread use of electronic medical record databases has enhanced patient safety through automation of signal detections for ADRs, thereby improving healthcare service delivery[13]. In Africa, the establishment and use of such databases is still rare [14] and ADR reporting is largely done manually. Strengthening of PV systems in sub-Saharan African (SSA) countries has received support from global health initiatives, but reporting is often siloed by disease specific (e.g. malaria, vaccines, HIV/AIDS) because of restricted funding streams rather than strengthening countrywide reporting systems[15]. As a result, PV systems in SSA remain weak[16]. In Uganda, 556 spontaneous reports were submitted to the National Pharmacovigilance Centre (NPC) in the initial five years of 2005-2009. Of these, 315 (57%) were related to medicines with 10 or more spontaneous ADR reports and were dominated by antiretroviral drugs (51%, 160/315), antimalarials (27%, 85/315), and antibiotics (22%, 70/315)[17]. The dominance of ADR reports related to these groups of medicines accords with the burden of disease in SSA[18].

The WHO's Uppsala Monitoring Centre (UMS) maintains web-based ADR reporting software (VigiFlow) for use by National Pharmacovigilance Centres[19]. Although receipt of 200 or more ADR reports per million population per year is desirable[20], most SSA countries submit fewer than 20 ADR reports per million population in 2010 compared to more than 100 reports per million in other low- and middle-income countries[21].

Uganda established a National Pharmacovigilance Centre (NPC) in 2005 and has been a member of the WHO program for International Drug Monitoring since 2007. In 2010, there was a training-of-trainers session for 30 national pharmacovigilance trainers. By 2011, 14 regional PV centres were established[21], PV-training sessions for core teams of healthcare professionals (HCPs) were conducted in



For peer review only - http://bmjopen.bmj.com/site/about/guidelines.xhtml

each of these centres, and ADR reporting forms distributed[22]. At least one support supervision visit per centre is conducted annually. Despite these efforts, reporting rate in Uganda (population: 36 million) is still low at 6 ADR reports per million population per year, based on 1,348 ADR reports in 2007-2012 [180, 75, 229[23], 140, 183, 413 in 2012 (when Targeted Spontaneous Reporting (TSR) was launched); and 128 in January-June 2013 (Nassali Huldah & Helen Ndagije, personal communication, 15 Jan 2014)]. Moreover, significant missing information in four-fifths of ADR reports compromises analysis[17].

Of 46 SSA countries whose PV systems were assessed to determine their capacity to ensure drug safety, Uganda was identified as one of four with active PV systems that could, in principle, detect, evaluate, and address medicine safety issues[24]. Indeed, Ugandan surgical series[25] on, and subsequent media coverage of, gluteal fibrosis and post injection paralysis among children injected with quinine[26, 27] triggered investigation by the Ugandan NPC which, in 2010, mediated change of Uganda's recommended quinine injection site from the gluteus muscle to the thigh[28].

Personal and professional characteristics associated with increased ADR reporting by HCPs include older age, male gender, lower workload, higher number of prescriptions issued per day, type of education received, specific PV training, and involvement in teaching and research[8, 29, 30]. Inhibitory factors include: unavailability of ADR forms, bureaucratic method of ADR reporting, and uncertainty over which professional cadre is mandated to report ADRs[31].

In 1996, Inman et al[32] described eight 'deadly sins' to explain why HCPs underreport ADRs: i) attitudes related to professional activities (financial incentives, fear of litigation, and ambition to publish personal case series), ii) ADR–related knowledge and attitudes (complacency, diffidence, indifference, and ignorance), and



For peer review only - http://bmjopen.bmj.com/site/about/guidelines.xhtml

iii) excuses made by HCPs (lethargy). Insecurity is an attitudinal factor that was not proposed by Inman but has been reported elsewhere[33].

In Africa, there is a paucity of empirical data on PV awareness[34-38]. Hence we sought to determine the level of PV awareness by HCPs, and the extent and determinants of past-month ADR recognition and of past-year ADR reporting in Uganda.

## Methods

### Study design and sampling procedure

From 25 May 2012 through 28 February 2013, we conducted a survey across Uganda in purposively selected, geographically diverse public and private health facilities. Public institutions included the National Referral Hospital-Mulago, and six Regional Referral Hospitals each selected to represent a major region of the country. In addition, we included District Hospitals and Health Centres (HCs) at levels II to IV in the catchment area where a Regional Referral Hospital was selected. For logistical reasons, we selected a convenience sample of private for-profit and private not-for-profit health facilities (which included drug shops) in the respective districts where public institutions were assessed. Permission to conduct the research was sought from the administrators of the selected institutions.

Any HCP involved in prescribing, transcribing, dispensing medication orders, and administration of drugs to a ~~hospital in~~patient was eligible for inclusion. Written informed consent was obtained from HCPs prior to their recruitment. The self-completed questionnaires did not contain identifying information on individual HCPs. The survey team used serial numbers to track distributed questionnaires. Five research assistants, all final year medical students at Mulago National Referral Hospital, were



For peer review only - http://bmjopen.bmj.com/site/about/guidelines.xhtml

initially recruited, and trained on the concepts of pharmacovigilance, informed consent, and response -rate and on the survey questionnaire which they by self-completed. ing it in an average of 27 minutes (22, 25, 27, 31, 31). Completion of questionnaire by research assistants was primarily to familiarize them with it and to gauge time to completion (22, 25, 27, 31 and 31; mean of 27 minutes) but served also as a brief pre-test. A similar model of data collection by pre-trained investigators was employed in the upcountry sites.

Formatted: Highlight

Given the challenge of accessing staff lists in the selected health facilities (and especially so in private-for-profit settings), random sampling of eligible HCPs was not practicable. Instead, in each health facility, the pre-trained investigators approached HCPs of all ranks and invited them to complete a pretested questionnaire, of which 2,200 were printed and 2,000 distributed. Invitations might be declined if HCPs were particularly busy or, despite willingness, a delay of several days or weeks might ensue before the self-completion questionnaire was returned. In practice, neither the refusal-rate by approached HCPs nor the 'did not return rate', by professional cadre, for distributed questionnaires was reliably documented.

In Uganda, there were reckoned to be 46,566 HCPs in 2009[39], who would have been survey-eligible had they worked at the survey-locations. Doctors and dentists (3,459) represented an estimated 7% of the nationally eligible staff but were 20% of the achieved sample; 762 pharmacists and pharmacy technicians 1.6% of nationally eligible staff but 6% of the achieved sample; and 37,625 nurses, midwives and nursing assistants an estimated 81% of the nationally eligible staff but 59% of the achieved sample.



For peer review only - http://bmjopen.bmj.com/site/about/guidelines.xhtml

**Data collection and management**

The survey questionnaire, see **Appendix**, elicited demographic and professional information, description of the most recent suspected ADR, and attitudes to, as well as knowledge and use of, the suspected ADR reporting system. The questionnaire for HCPs included 15 attitudinal statements on ADR reporting which were to be scored from 1 (total disagreement) to 5 (total agreement). All data were entered into a databank using EpiData 3.1.

Prior to its administration, the questionnaire was elaborated between members of the research team who have diverse expertise in pharmacy, pharmacovigilance, and questionnaire design. Completion-time was tested by research assistants. Thereafter, an integrated pilot study was conducted on 125 healthcare professionals. The subsequent revisions were sufficiently minor that results of the pre-test were included in the final analysis.

The questionnaire was initially tested on 125 participants. The subsequent revisions sufficiently minor that results of the pre-test were included in the final analysis.

Formatted: Font: Times New Roman, Not Highlight

Formatted: Font: Times New Roman

Formatted: Font: Times New Roman, Not Highlight

Formatted: Font: Times New Roman

Formatted: Font: Times New Roman, Not Highlight

Formatted: Font: Times New Roman

**Statistical analysis**

Responses are summarized as frequencies and percentages. Different potential determinants for the past-month recognition or past-year reporting of suspected ADRs were screened using $\chi^2$-tests for categorical variables. Logistic regression was then used to assess the relationship of demographic and professional factors severally to: i) recognition of suspected ADRs in the past 4 weeks; and for those in post for at least one year, ii) having reported at least one suspected ADR in the past 12 months. Attitudinal factors were also incorporated in ii). Missing data were accounted for using multiple imputations under the missing at random assumption[40] on the one hand



For peer review only - http://bmjopen.bmj.com/site/about/guidelines.xhtml

or, as here, the missing-assigned approach on the other, where missing data were meaningfully assigned to an existing category. Results are expressed as odds ratios (ORs) with 95% confidence intervals. Statistical analyses were carried out using Stata 12.0[41].

**Ethical clearance**

Ethical approval was obtained from the School of Medicine Research and Ethics Committee, Makerere University College of Health Sciences, and the Uganda National Council for Science and Technology.



For peer review only - http://bmjopen.bmj.com/site/about/guidelines.xhtml

## Results

### Study population

Of 2,000 questionnaires distributed, 1,345 were completed, a return-rate of 67%. Mean age of respondent HCPs was 32.4 years (SD = 8.9). Nurses were the majority (776/1,340 or 58%), see **Table 1**.

### Awareness of pharmacovigilance

Half the respondents (678/1,310 or 52%; 95% CI: 49% to 55%) had ever heard about pharmacovigilance: two-fifths of nurses (295/763 or 39%; 95% CI: 35% to 42%) but 70% of others (383/547; 95% CI: 66% to 74%). Thirty percent of HCPs (412/1,317; 95% CI: 29% to 34%) were aware of the existence of Uganda's NPC but only 3% (37/1,312; 95% CI: 2% to 4%) of HCPs had *ever* submitted an ADR report to the NPC.

### Suspected ADR reporting in the previous 12 months

Only 15% of HCPs (190/1,296; 95% CI: 13% to 17%) had reported a suspected ADR in the previous 12 months, of whom 15% (27/175) claimed to have made their report to NPC so that our respondents' past-year ADR reporting rate to NPC was an estimated 1 in 50 (2%). Only 41% (11/27; 95% CI: 22% to 59%) past-year reporters to NPC had found the NPC-form clear on what to report.

When HCPs were asked about when, in the past 12 months, they had reported their most recent suspected ADR, 79/178 (44%) said within the past month, 28 (16%) in the months 2+3 prior, and 71 (40%) in months 4-12, a distribution indicative either of a multiplicity of reports per ADR-reporter or biased recall.



For peer review only - http://bmjopen.bmj.com/site/about/guidelines.xhtml

**ADR recognition**

Twenty one per cent (268/1,289: 95% CI, 19% to 23%) of respondents had suspected an ADR in the previous one month, 76% of whom (195/257: 95% CI, 71% to 81%) had received patient ADR-complaints in the past month. Of HCPs who had suspected an ADR in the past month, 35% (92/262: 95% CI, 29% to 41%) had reported an ADR in the past 12 months.

Among HCPs who had not suspected an ADR in the previous month, 12% (121/1,000: 95% CI, 10% to 14%) had nonetheless received patient ADR-complaints in the past month.

In the previous 4 weeks, see **Table 2**, 26% (340/1,302) of HCPs had received 1,190 patient ADR-complaints [mean of 3.5 complaints (sd 9.5) per complaint-receiving HCP] which equates to 0.9 ADR-complaints (95% CI: 0.65 to 1.18) per HCP per month. Also, 21% (268/1,289) of HCPs had suspected 670 ADRs [mean of 2.5 suspected ADRs (sd 2.6) per suspecting HCP] which equates to 0.5 suspected ADRs (95% CI: 0.45 to 0.59) per HCP per month, implying an ADR suspicion rate of 0.57 (0.52/0.91) per patient ADR-complaint per HCP per month (95% CI: 0.42 to 0.80).

Among the 15% (190/1,296) who were ADR-reporters in the previous 12 months, 44% (79/178) claimed to have submitted their most recent report in the past 4 weeks. If so, there could be at least 84 suspected ADR reports submitted by 1,296 HCPs in the past 4 weeks (or 0.065 ADR-reports in past 4 weeks per HCP) when 0.5 ADRs were suspected in the past 4 weeks per HCP. This translates into a 13% ADR-report rate per suspected ADR.

**Medication classes and fatalities in survey-described suspected ADRs**



For peer review only - http://bmjopen.bmj.com/site/about/guidelines.xhtml

The most frequently mentioned medication classes associated with 182 survey-described ADRs in the past 4-weeks which cited one or more drugs (216 drug citations) were antibiotics (38%, 83/216), antiretroviral agents (23%, 49/216), antimalarials (15%, 33/216, 15 of which implicated quinine), analgesics (9%, 19/216), and others (15%, 32/216).

Two suspected ADRs were described by HCPs which involved child fatalities in association with quinine: a 5-year-old girl had been given intravenous quinine and died soon after arrival at a private-not-for-profit hospital in Eastern Uganda; and a 2-year old boy had reacted to quinine and died despite the doctor in a public hospital in Eastern Uganda having administered an antidote. Full details of HCPs' described suspected ADRs will be reported separately.

**Feedback to ADR reporters**

Reporters of ADRs to AIDS Treatment Information Centre (ATIC) received the highest feedback (60%, 12/20), followed by those who reported to the Medical Superintendent or Institutional Review Board (39%: 23/58 + 4/11). Feedback from Uganda's NPC was infrequent (23%: 5/22). Reporters of ADRs to drug manufacturers (4) or District Directors of Health Services (12) received zero feedback.

**Reasons for ADR reporting**

The commonest reason that respondents vouched for ADR reporting was that the patient had developed a serious ADR (30%, 48/159 reasons) followed by patient safety (18%, 29/159), and patient ADR-complaint (8%, 13/159). The next three reasons each had nine citations: institutional mandate to report ADRs, prevention of similar ADRs, and as a means of obtaining advice.



For peer review only - http://bmjopen.bmj.com/site/about/guidelines.xhtml

**Attitudes to ADR reporting**

Only 14% (186/1,301: 95% CI, 12% to 16%) of respondents indicated that reporting ADRs put their career at risk, see **Table 3**, while 36% (466/1,304: 95% CI, 33% to 38%) thought that it is only necessary to report serious or unexpected ADRs. Most respondents agreed that they have a professional obligation to report ADRs (76%, 1,000/1,311: 95% CI, 74% to 79%) and 68% (896/1,319: 95% CI, 65% to 70%) stated that they would report ADRs if there were an easier method. Forty five per cent (596/1,312: 95% CI, 43% to 48%) stated that they do not know how information reported in the ADR form is used, 64% (833/1,309: 95% CI, 61% to 66%) felt that they would report an ADR only if they were sure it was related to use of a particular drug, and 27% (349/1,305: 95% CI, 24% to 29%) felt that they should be financially reimbursed for providing the ADR reporting service.

Formatted: Space After: 12 pt

**Factors associated with ADR suspicion in the past month**

Suspicion of ADR in the past 4-weeks was more likely by non-nurses (odds ratio (OR) = 1.7, 95% CI: 1.16 – 2.40) and with involvement in medical research (OR = 1.5, 95% CI: 1.05 – 2.15) but the clearly dominant factor was that the HCP had received patient ADR-complaint(s) in the past 4-weeks (OR = 19, 95% CI: 14-28). There was some evidence that ADR suspicion was less likely by staff in surgical wards, see **Table 4**.

Logistic regression analysis among the 973 respondents who did not receive a patient ADR complaint did not identify any additional significant cofactors associated with ADR suspicion.

Formatted: Space After: 12 pt

**Personal, professional and attitudinal factors associated with having made an ADR report in the past 12 months**



For peer review only - http://bmjopen.bmj.com/site/about/guidelines.xhtml

Demographic and professional factors associated with a lower likelihood to report ADRs in the past 12 months were: private for-profit health facility (vs. public; OR = 0.5, 95% CI: 0.28 – 0.77) and HCP aged over 30 years (OR = 0.6, 95% CI: 0.43 – 0.91); while those associated with being more likely to report ADRs included: medical department (vs. surgery; OR = 2.3, 95% CI: 1.08 – 4.73), having ever encountered a fatal ADR (OR = 2.9, 95% CI: 1.94 – 4.25), knowing to whom to report ADRs (OR = 1.7, 95% CI: 1.18 – 2.46), and HCPs who had suggested ways of improved ADR reporting (OR = 1.6, 95% CI: 1.04 – 2.49), see **Table 5.**

Only two attitudinal factors were additionally relevant: diffidence ('the belief that reporting an ADR would only be done if there was certainty that it was related to the use of a particular drug'; OR = 0.6, 95% CI: 0.41-0.89) and lethargy ('I do not know how information reported in ADR form is used'), see **Table 6**.

**Suggestions for improved ADR reporting**

The most frequently cited suggestion was to sensitize, train and provide ongoing medical education on ADRs to HCPs (42%, 667/1,589 suggestions) followed by making ADR forms available (17%, 262/1,589), sensitizing the public and counselling patients about ADRs (11%, 166/1,589), creating a coordinating office in each health facility (5%, 73/1,589), providing financial incentives to reporters (4%, 65/1,589), and making available telephone or online ADR reporting systems (4%, 57/1,589), see **Table 7**.



For peer review only - http://bmjopen.bmj.com/site/about/guidelines.xhtml

## Discussion

A low proportion of HCPs reported having submitted an adverse drug reaction (ADR) report in the previous 12 months (15%) and the level of awareness of PV was also low, similar to observations made elsewhere[34, 42, 43]. Healthcare professionals from different cadres may recognize suspected ADRs but fail to take the responsibility to report[44]. Barely one in eight (13%) of suspected ADRs in the past month was reported by the HCPs in that same period, yet around three-fifths of patient ADR-complaints in the past month were adjudged by HCPs to be suspected ADRs. Integration of pharmacovigilance into pre-service training curricula and emphasizing its importance in promoting patient safety in healthcare delivery is a first step[45, 46] upon which other PV initiatives can build.

To raise the number of submitted ADR reports, Uganda has proposed mandatory reporting of ADRs by industry and HCPs[22]. However, questions have been raised about the effectiveness of compulsory reporting by HCPs[47] and the NPC needs to improve its feedback to ADR reporters since our respondents ranked it much lower than ATIC. Moreover, HCPs in our study reported ADRs to a greater extent than in nationally-reported statistics: 2% of HCPs (27/1281: 95% CI, 1.3% to 2.9%) had reported any suspected ADR to the NPC in the previous year compared with the NPC's annual average national ADR reporting rate for Uganda from 2007 to mid-2013 of 0.44% [based on 1,348 reports in 6.5 years from 46,566 clinical staff countrywide: 95% CI, 0.38% to 0.51%] or 0.90% in the highest report-year of 2012 [413 reports in 2012: 95% CI, 0.80% to 0.97%]. Thus, HCPs in our study seemed at least twice as likely to have submitted suspected ADRs to the NPC in the previous year when compared with the national ADR reporting rates by Uganda's HCPs.



For peer review only - http://bmjopen.bmj.com/site/about/guidelines.xhtml

One limitation to our estimates is that more than one HCP may have described (and reported) the same suspected ADR since our ability to discriminate between suspected ADRs was compromised by variation in the quality of ADR descriptions, a limitation that NPC also contends with.

Consistent with ADR reports from the NPC[17], we identified antibiotics, antiretroviral agents, and antimalarials as the three most frequently cited medication classes in survey-described ADRs. Therefore, health initiatives already focusing on the PV of these medications, if replicated for other classes, present opportunities to strengthen overall PV systems in these settings[17]. As a PV exemplar in Uganda, the NPC and AIDS Control Program introduced TSR in 2011 to monitor tenofovir for renal toxicity and to detect suspected ADRs related to antiretroviral therapy use in the Prevention of Mother to Child Transmission of HIV and in the Early Infants Diagnosis program[48]. Results from TSR are yet to be disseminated, however.

Around three-fifths of patients' ADR-complaints to HCPs in the past month translated into ADR suspicion. Patient ADR-complaint was dominant among explanatory factors for HCPs' ADR-suspicion in the past month and so we suggest that empowering patients to support HCPs may improve the detection and reporting of suspected ADRs. Moreover, other countries have instituted systems that promote spontaneous direct patient reporting of suspected ADRs thus permitting patients to participate in PV activities that teach them to handle their medicines better and improves their communication with HCPs[49, 50].

Improvement of the ADR reporting form for Uganda seems necessary. Therefore, our research team designed a form that is relevant to the inpatient setting and captures additional information required for causality assessment of suspected medicines. This form will be tested in a follow-up study on inpatients.



For peer review only - http://bmjopen.bmj.com/site/about/guidelines.xhtml

Other suggestions to improve ADR reporting by respondents included; increased visibility of the NPC and giving useful feedback to ADR reporters, introducing telephone and online reporting systems, increasing onsite support supervision, making ADR forms more available, providing training and continued medical education of HCPs as suggested elsewhere[51], and sensitizing the public to ADRs. The absence of a national PV policy, however, coupled with the lack of proper coordination between the NPC and numerous health programmes and sentinel sites may undermine efforts to strengthen the countrywide PV system[17]. For example, in Uganda's teaching hospitals, could some clinical grand rounds address PV and suspected serious ADRs?

Although previous studies suggested a positive relationship between older age and ADR reporting[52, 53], we found that older HCPs (≥30 years) were less likely than their younger counterparts to have reported suspected ADRs in the past 12 months. These contrasting results might be attributed to idiosyncratic differences between HCPs and healthcare systems in Europe and Africa such that younger staff, as in our study, may have had more PV training. There is, as yet, limited published literature from other African settings. Our respondents were, on average, 10 years younger when compared with studies conducted in Europe[29]. We suggest that older HCPs in Uganda be targeted in future strategies on improved ADR reporting.

In contrast to other studies[53], training on how to report ADRs was not significantly associated with increased ADR reporting. Given the cross-sectional study design we used, it was not possible to establish whether PV training preceded ADR reporting, or vice versa, and therefore we were unable to assess their temporal relationship. That notwithstanding, Gonzalez-Gonzalez *et al* have suggested that



For peer review only - http://bmjopen.bmj.com/site/about/guidelines.xhtml

multifaceted interventions, as opposed to single educational programmes, increase to a greater extent HCPs' PV awareness and motivate them to report ADRs[8].

A low level of PV awareness may lead to underreporting of ADRs[54]. In our study, knowing to whom to report was an important factor for ADR reporting in the final logistic regression. We also observed that the proportion (31%: 95% CI, 29% to 34%) of respondents aware of the existence of Uganda's NPC is lower than reported for Nigeria [52% (51/99): 95% CI, 42% to 61%][34]. Much higher proportions of PV awareness have been reported in Europe[29] and Asia[55, 56] where there are higher ADR reporting rates per million of population[57] and more government involvement in national PV programs[34].

Healthcare professionals who had ever encountered a fatal ADR were twice as likely to report an ADR as HCPs who had not. Correspondingly, development of a serious or fatal ADR was the most frequently cited reason for ADR reporting. We also found that HCPs who suggested possible ways of improving the ADR reporting system were more likely to have reported an ADR in the previous 12 months[58].

Healthcare professionals who agreed with the statement 'I would only report an ADR if I was sure that it was related to the use of a particular drug' (diffidence) were less likely to report suspected ADRs. Apart from diffidence and lethargy/indifference ('I do not know how information reported in the ADR form is used'), none of the other Inman factors was associated with ADR reporting[8, 32, 59]. Diffidence and lethargy can be targeted in educational interventions to promote ADR reporting and by improved feedback to ADR-reporters.

Although provision of financial incentives to reporters was the fifth most frequently cited suggestion to improve ADR reporting, it was not statistically



For peer review only - http://bmjopen.bmj.com/site/about/guidelines.xhtml

significant in the logistic regression for the odds on ADR reporting and these findings are consistent with those in the developed world[60].

In private for-profit health facilities, HCPs were less likely to have reported ADRs in the previous 12 months than their counterparts in the public sector. In addition, HCPs in hospitals (public and private) were twice as likely as those from other health facilities (HCs II & III, community pharmacies, drug shops) to have reported suspected ADRs in the previous 12 months. Whereas few PV scale-up activities in Africa have given priority to the private sector[16, 22], more public-private collaboration could strengthen PV systems in our SSA setting[61].

Our study had several limitations. First, we used self-report as the main method of inquiry and this may have introduced recall bias. Second, we may have experienced social desirability bias as HCPs may not have given frank responses for fear of being embarrassed if they were not reporting ADRs. However, as we used self-administered questionnaires without respondents' names, the potential for this bias was reduced. Third, the cross-sectional design that we used could not establish temporal relationships between ADR reporting in past year and some explanatory factors. Fourth, there was over-representation of doctors and pharmacists/pharmacy technicians versus nurses. Finally, several respondents may have referred to the same suspected ADR but this did not have a significant bearing since our main focus was assessment of individual ADR reporting behaviour rather than on individual ADRs.

Our study has, however, generated key insights on determinants in Uganda for HCPs' ADR suspicion and reporting.

## Conclusions

One in five HCPs had suspected an ADR in the past 4 weeks while one in seven had reported an ADR in the previous 12 months. Empowering patients to



For peer review only - http://bmjopen.bmj.com/site/about/guidelines.xhtml

support HCPs in suspected ADR detection and reporting is essential to strengthening PV systems in Africa. HCPs who ever encountered fatal ADRs are keener reporters and can consequently help others to avoid the experience that made them better reporters. HCPs ought to know that they don't have to be certain about causality to report suspected ADRs. Poor access to suspected ADR forms and lack of feedback on reports are constraints that can be rectified. [4,279 words]

## Funding

This work was supported by Training Health Researchers into Vocational Excellence (THRiVE) in East Africa, grant number 087540, funded by the Wellcome Trust; grant number 5R24TW008886 supported by OGAC, NIH and HRSA; and an African Doctoral Dissertation Research Fellowship award offered by the African Population and Health Research Centre (APHRC) in partnership with the International Development Research Centre (IDRC). In UK, SMB is funded by Medical Research Council programme number MC_U105260794.

## Role of funders

The work here reported is solely the responsibility of the authors and does not necessarily represent the official views of the supporting offices.

The funders had no role in the decisions on what and where to publish.

## Competing Interests

SMB holds GSK shares. The authors declare that they otherwise have no competing interests.



For peer review only - http://bmjopen.bmj.com/site/about/guidelines.xhtml

## Authors' contributions

RK conceived of the study and drafted the manuscript and, in conjunction with SMB, participated in its design, implementation, statistical analysis and the drawing of inferences. CK, PW and HBN participated in study design and in the process of manuscript writing. All authors approved the final manuscript.


## Acknowledgements

The authors wish to thank all the HCPs who agreed to participate in this study.

We also thank Huldah Nassali at the National Pharmacovigilance Centre for providing technical support to this project.

Finally, RK thanks Noeline Nakasujja at Makerere University College of Health Sciences, and Yukari C. Manabe at Johns Hopkins University for supporting his development of the first three drafts of the manuscript.


For peer review only



For peer review only - http://bmjopen.bmj.com/site/about/guidelines.xhtml

References

1. Lazarou J, Pomeranz BH, Corey PN. Incidence of adverse drug reactions in hospitalized patients: a meta-analysis of prospective studies. *JAMA* 1998; **279**(15): 1200-5.
2. Ramesh M, Pandit J, Parthasarathi G. Adverse drug reactions in a south Indian hospital--their severity and cost involved. *Pharmacoepidemiol Drug Saf* 2003; **12**(8): 687-92.
3. Classen DC, Pestotnik SL, Evans RS, Lloyd JF, Burke JP. Adverse drug events in hospitalized patients. Excess length of stay, extra costs, and attributable mortality. *JAMA* 1997; **277**(4): 301-6.
4. Bates DW, Spell N, Cullen DJ, et al. The costs of adverse drug events in hospitalized patients. Adverse Drug Events Prevention Study Group. *JAMA* 1997; **277**(4): 307-11.
5. Gautier S, Bachelet H, Bordet R, Caron J. The cost of adverse drug reactions. *Expert Opin Pharmacother* 2003; **4**(3): 319-26.
6. World Health Organization. The Safety of Medicines in Public Health Programmes: Pharmacovigilance an Essential Tool. 2006. http://www.who.int/medicines/areas/quality_safety/safety_efficacy/Pharmacovigilance_B.pdf (accessed 31 May 2014).
7. Lexchin J. Is there still a role for spontaneous reporting of adverse drug reactions? *CMAJ : Canadian Medical Association journal = journal de l'Association medicale canadienne* 2006; **174**(2): 191-2.
8. Lopez-Gonzalez E, Herdeiro MT, Figueiras A. Determinants of under-reporting of adverse drug reactions: a systematic review. *Drug Saf* 2009; **32**(1): 19-31.
9. Hazell L, Shakir SA. Under-reporting of adverse drug reactions : a systematic review. *Drug Saf* 2006; **29**(5): 385-96.
10. Smith CC, Bennett PM, Pearce HM, et al. Adverse drug reactions in a hospital general medical unit meriting notification to the Committee on Safety of Medicines. *Br J Clin Pharmacol* 1996; **42**(4): 423-9.
11. Pirmohamed M, Breckenridge AM, Kitteringham NR, Park BK. Adverse drug reactions. *BMJ* 1998; **316**(7140): 1295-8.
12. Gavaza P, Brown CM, Lawson KA, Rascati KL, Wilson JP, Steinhardt M. Influence of attitudes on pharmacists' intention to report serious adverse drug events to the Food and Drug Administration. *Br J Clin Pharmacol* 2011; **72**(1): 143-52.
13. Miguel A, Azevedo LF, Lopes F, Freitas A, Pereira AC. Methodologies for the detection of adverse drug reactions: comparison of hospital databases, chart review and spontaneous reporting. *Pharmacoepidemiol Drug Saf* 2013; **22**(1): 98-102.
14. Alj L, Touzani MDW, Benkirane R, Edwards IR, Soulaymani R. Detecting medication errors in pharmacovigilance database: Capacities and limits. *Int J Risk Saf Med* 2007; **19**: 187-94.
15. Strengthening Pharmaceutical Systems (SPS). Supporting Pharmacovigilance in Developing Countries: The Systems Perspective
Arlington, VA: Submitted to the U.S. Agency for International Development by the SPS Program, 2009.
16. Isah AO, Pal SN, Olsson S, Dodoo A, Bencheikh RS. Specific features of medicines safety and pharmacovigilance in Africa. *Therapeutic Advances in Drug Safety* 2012; **3**(1): 25-34.



For peer review only - http://bmjopen.bmj.com/site/about/guidelines.xhtml

17. National Pharmacovigilance Centre. Working together with the Public Health Programmes: a regulator's perspective for addressing the minimum requirements for Pharmacovigilance. 2010. http://www.who.int/medicines/areas/quality_safety/regulation_legislation/icdra/WG-2_2Dec.pdf (accessed 21 June 2013).
18. Institute for Health Metrics and Evaluation. The Global Burden of Disease: Generating Evidence, Guiding Policy-Sub-Saharan Africa Regional Edition. 2013. http://www.healthdata.org/policy-report/global-burden-disease-generating-evidence-guiding-policy%E2%80%94sub-saharan-africa-regional (accessed 31 May 2014).
19. Uppsala Monitoring Centre (UMC). VigiFlow ™ The complete Individual Case Safety Report (ICSR) management system from the UMC. http://www.who-umc.org/graphics/27705.pdf (accessed 31 May 2014).
20. Uppsala Monitoring Centre (UMC). Pharmacovigilance - Reporting Trends. 2013. http://who-umc.org/DynPage.aspx?id=108476&mn1=7347&mn2=7252&mn3=7322&mn4=7558 (accessed 31 May 2014).
21. Strengthening Pharmaceutical Systems (SPS) Program. Safety of Medicines in sub-Saharan Africa: Assessment of Pharmacovigilance Systems and their Performance. 2011. http://apps.who.int/medicinedocs/documents/s19152en/s19152en.pdf2014).
22. Ouma C, Abwao E. Africa Pharmacovigilance Meeting 2012: Technical Report. Submitted to the US Agency for International Development by the Systems for Improved Access to Pharmaceuticals and Services Program, 2012.
23. National Pharmacovigilance Centre. The Pharmacovigilance News. 2010. http://www.nda.or.ug/docs/bulletin%20issue%20No.1.pdf (accessed 1 June 2014).
24. Appiah B. Africa struggles to improve drug safety. *CMAJ : Canadian Medical Association journal = journal de l'Association medicale canadienne* 2012; **184**(10): E533-4.
25. Ekure J. Gluteal Fibrosis. A report of 28 cases from Kumi Hospital, Uganda. *East and Central African Journal of Surgery* 2006; **12**(1): 144-7.
26. Ekure J. Post-injection paralysis: The new polio in Uganda. 9 December 2007. http://www.newvision.co.ug/D/9/34/601055.
27. Chinnock P. Concern in Uganda over children injured by poorly administered malaria treatment 2009. http://www.tropika.net/svc/news/20090604/Chinnock-20090604-News-Quinine-Uganda (accessed 13 May 2014).
28. National Pharmacovigilance Centre. What is current practice of Pharmacovigilance in Uganda? 2012. http://africapv2012.files.wordpress.com/2012/04/day-2_5_country-presentations-nras_h-ngadije-uganda-compatibility-mode.pdf (accessed 11 December 2013).
29. Bateman DN, Sanders GL, Rawlins MD. Attitudes to adverse drug reaction reporting in the Northern Region. *Br J Clin Pharmacol* 1992; **34**(5): 421-6.
30. Figueiras A, Tato F, Fontainas J, Gestal-Otero JJ. Influence of physicians' attitudes on reporting adverse drug events: a case-control study. *Med Care* 1999; **37**(8): 809-14.
31. Rawlins MD. Pharmacovigilance: paradise lost, regained or postponed? The William Withering Lecture 1994. *J R Coll Physicians Lond* 1995; **29**(1): 41-9.
32. Inman WH. Attitudes to adverse drug reaction reporting. *Br J Clin Pharmacol* 1996; **41**(5): 434-5.



For peer review only - http://bmjopen.bmj.com/site/about/guidelines.xhtml

33. Herdeiro MT, Figueiras A, Polonia J, Gestal-Otero JJ. Physicians' attitudes and adverse drug reaction reporting : a case-control study in Portugal. *Drug Saf* 2005; **28**(9): 825-33.
34. Oshikoya KA, Awobusuyi JO. Perceptions of doctors to adverse drug reaction reporting in a teaching hospital in Lagos, Nigeria. *BMC Clin Pharmacol* 2009; **9**: 14.
35. Robins AH, Weir M, Biersteker EM. Attitudes to adverse drug reactions and their reporting among medical practitioners. *South African medical journal = Suid-Afrikaanse tydskrif vir geneeskunde* 1987; **72**(2): 131-4.
36. Ball D, Tisocki T. Adverse drug reaction reporting by general medical practitioners and retail pharmacists in Harare--a pilot study. *Cent Afr J Med* 1998; **44**(8): 190-5.
37. Okezie EO, Olufunmilayo F. Adverse drug reactions reporting by physicians in Ibadan, Nigeria. *Pharmacoepidemiol Drug Saf* 2008; **17**(5): 517-22.
38. Jaquet A, Djima MM, Coffie P, et al. Pharmacovigilance for antiretroviral drugs in Africa: lessons from a study in Abidjan, Cote d'Ivoire. *Pharmacoepidemiol Drug Saf* 2011; **20**(12): 1303-10.
39. Africa Health Workforce Observatory. Human Resources for Health Country Profile - Uganda. 2009. p. 30.
40. Sterne JA, White IR, Carlin JB, et al. Multiple imputation for missing data in epidemiological and clinical research: potential and pitfalls. *BMJ* 2009; **338**: b2393.
41. StataCorp. Stata Statistical Software: Release 12. College Station, TX: StataCorp LP; 2011.
42. Khan SA, Goyal C, Chandel N, Rafi M. Knowledge, attitudes, and practice of doctors to adverse drug reaction reporting in a teaching hospital in India: An observational study. *Journal of natural science, biology, and medicine* 2013; **4**(1): 191-6.
43. Pimpalkhute SA, Jaiswal KM, Sontakke SD, Bajait CS, Gaikwad A. Evaluation of awareness about pharmacovigilance and adverse drug reaction monitoring in resident doctors of a tertiary care teaching hospital. *Indian journal of medical sciences* 2012; **66**(3-4): 55-61.
44. Bemt van den PMLA, Egberts ACG. Drug-related problems: definitions and classifications. *EJHP* 2007; (13): 62-4.
45. Gallagher TH, Waterman AD, Ebers AG, Fraser VJ, Levinson W. Patients' and physicians' attitudes regarding the disclosure of medical errors. *JAMA* 2003; **289**(8): 1001-7.
46. Cox AR, Marriott JF, Wilson KA, Ferner RE. Adverse drug reaction teaching in UK undergraduate medical and pharmacy programmes. *J Clin Pharm Ther* 2004; **29**(1): 31-5.
47. Cross C. Drug reporting may be mandatory for doctors. *CMAJ : Canadian Medical Association journal = journal de l'Association medicale canadienne* 2013; **185**(10): 866.
48. Uppsala Monitoring Centre. Uppsala Reports 59. 2012.
49. Andrew Herxheimer, Rose Crombag, Teresa Leonardo Alves. Direct Patient Reporting of Adverse Drug Reactions. May 2010.
50. Masso Guijarro P, Aranaz Andres JM, Mira JJ, Perdiguero E, Aibar C. Adverse events in hospitals: the patient's point of view. *Quality & safety in health care* 2010; **19**(2): 144-7.
51. Bukirwa H, Nayiga S, Lubanga R, et al. Pharmacovigilance of antimalarial treatment in Uganda: community perceptions and suggestions for reporting adverse events. *Trop Med Int Health* 2008; **13**(9): 1143-52.

**Formatted:** Font color: Red



For peer review only - http://bmjopen.bmj.com/site/about/guidelines.xhtml

52. Tubert-Bitter P, Haramburu F, Begaud B, Chaslerie A, Abraham E, Hagry C. Spontaneous reporting of adverse drug reactions: who reports and what? *Pharmacoepidemiol Drug Saf* 1998; **7**(5): 323-9.
53. Irujo M, Beitia G, Bes-Rastrollo M, Figueiras A, Hernandez-Diaz S, Lasheras B. Factors that influence under-reporting of suspected adverse drug reactions among community pharmacists in a Spanish region. *Drug Saf* 2007; **30**(11): 1073-82.
54. Ohaju-Obodo JO, Iribhogbe OI. Extent of pharmacovigilance among resident doctors in Edo and Lagos states of Nigeria. *Pharmacoepidemiol Drug Saf* 2010; **19**(2): 191-5.
55. Li Q, Zhang SM, Chen HT, et al. Awareness and attitudes of healthcare professionals in Wuhan, China to the reporting of adverse drug reactions. *Chin Med J (Engl)* 2004; **117**(6): 856-61.
56. Chatterjee S, Lyle N, Ghosh S. A survey of the knowledge, attitude and practice of adverse drug reaction reporting by clinicians in Eastern India. *Drug Saf* 2006; **29**(7): 641-2.
57. Aagaard L, Strandell J, Melskens L, Petersen PS, Holme Hansen E. Global patterns of adverse drug reactions over a decade: analyses of spontaneous reports to VigiBase. *Drug Saf* 2012; **35**(12): 1171-82.
58. Passier A, ten Napel M, van Grootheest K, van Puijenbroek E. Reporting of adverse drug reactions by general practitioners: a questionnaire-based study in the Netherlands. *Drug Saf* 2009; **32**(10): 851-8.
59. Herdeiro MT, Figueiras A, Polonia J, Gestal-Otero JJ. Influence of pharmacists' attitudes on adverse drug reaction reporting : a case-control study in Portugal. *Drug Saf* 2006; **29**(4): 331-40.
60. Herdeiro MT, Polonia J, Gestal-Otero JJ, Figueiras A. Factors that influence spontaneous reporting of adverse drug reactions: a model centralized in the medical professional. *J Eval Clin Pract* 2004; **10**(4): 483-9.
61. World Health Organization (WHO) - MMV joint technical consultation on active pharmacovigilance monitoring with a special focus on affordable medicine facility for malaria (AMFm). Pharmacovigilance Guidance for Countries Participating in AMGm Phase 1. 2009. http://apps.who.int/medicinedocs/documents/s18427en/s18427en.pdf (accessed 1 June 2014).



For peer review only - http://bmjopen.bmj.com/site/about/guidelines.xhtml

## Appendix

| Assessment of Adverse Drug Reaction Reporting among Healthcare Professionals in Uganda | |
|---|---|
| **Investigator:** ________________ | **District:** ________________ |
| An **Adverse Drug Reaction** (ADR) is any response to a drug which is harmful and unintended, and which occurs at doses normally used by patients. | |
| **HEALTH FACILITY CHARACTERISTICS** | |
| **1. Type of health facility** (*Tick one only*)<br>[1] Public<br>[2] Private Not-for-Profit<br>[3] Private For-Profit | **2. Level of health facility** (*Tick one only*)<br>[1] National Referral [5] Health Centre III<br>[2] Regional Referral [6] Health Centre II<br>[3] District Hospital [7] Private Hospital<br>[4] Health Centre IV [8] Other………….... |
| **SOCIO-DEMOGRAPHIC CHARACTERISTICS OF PARTICIPANT** | |
| **3. Gender**<br>[1] Male<br>[2] Female | **4. How old are you (in complete years)?** ……….. |
| **PROFESSIONAL CHARACTERISTICS OF PARTICIPANT** | |
| **5. In which sector(s) do you practice**? (*Tick all that apply*)<br>[1] Public health facility<br>[2] Private Not-for-Profit health facility<br>[3] Private For-Profit health facility | **6. In which department are you?** (*Tick one only*)<br>[1] Medicine<br>[2] Surgery<br>[3] Paediatrics<br>[4] Obstetrics & Gynaecology<br>[5] Dentistry<br>[6] Pharmacy<br>[7] Other (Specify)……….. |
| **7. What is the approximate number of patients you see per day**? …………… | **8. For how long have you been working in this health facility?** ....... Months (If less than 1 year)<br>….. Completed Years |
| **9. What is your highest academic qualification?** (*Tick one only*)<br>[1] Certificate<br>[2] Diploma<br>[3] First Degree<br>[4] Masters Degree<br>[5] PhD | **10. For how long have you been practicing since you qualified with your highest academic training?**<br>…… Months (If less than 1 year)<br>…… Completed Years |
| **11. Do you teach medical students?**<br>[1] Yes [2] No (If no, **go to 13**) | **12. If yes, duration of practice in a teaching hospital**<br>…… Months (If less than 1 year)<br>…… Completed Years |

For peer review only

| | |
|---|---|
| **13. Are you actively involved in medical research?**<br><br>[1] Yes [2] No | **14. Professional Cadre** (*Tick one only*)<br><br>[1] Doctor (**go to 15**)<br>[2] Pharmacist (**go to 22**)<br>[3] Nurse (**go to 19**)<br>[4] Clinical officer (**go to 23**)<br>[5] Pharmacy Technician (**go to 22**)<br>[6] Other (Specify)……………………… |
| **15. Position/Level of Doctor** (*Tick one only*)<br><br>[1] Senior Consultant<br>[2] Consultant<br>[3] Medical Officer Special Grade<br>[4] Medical Officer<br>[5] Senior House Officer<br>[6] Intern Doctor<br>[7] Other (specify)…………. | **16. For how long have you been prescribing?**<br><br>…… Months (If less than 1 year)<br>…… Completed Years |
| **17. What is the approximate number of prescriptions you write per day?**............. | **18. Have you given verbal prescriptions/orders to the attending nurse in the past 12 months?**<br><br>[1] Yes [2] No<br>(***Skip to 23***) |
| **19. Which of the following cadre category describes your qualification?** (*Tick one only*)<br><br>[1] Enrolled Midwife<br>[2] Enrolled Nurse<br>[3] Enrolled Mental Health Nurse<br>[4] Enrolled Comprehensive Nurse<br>[5] Registered Midwife<br>[6] Registered Nurse<br>[7] Registered Nurse/Midwife<br>[8] Registered Mental Health Nurse<br>[9] Registered Comprehensive Nurse<br>[10] Other (specify)…………………… | **20. In some health facilities, nurses usually write out (transcribe) drug prescriptions from patients' medical records to medication charts. Are you required to transcribe prescriptions in your health facility?**<br><br>[1] Yes [2] No |
| **21. In practice, do you regularly transcribe prescriptions?**<br><br>[1] Yes [2] No<br>(***Skip to 23***) | **22. If pharmacist or pharmacy technician, area of practice** (*Tick all that apply*)<br><br>[1] Hospital [3] Academia<br>[2] Industry [4] Community/Private |

**SUSPECTED ADVERSE DRUG REACTION (ADR) REPORTING PROGRAM**

For peer review only

| | |
|---|---|
| **23. Have you received any complaint of adverse drug reactions (ADRs) from patients in the last 4 weeks?**<br><br>[1] Yes [2] No (If no, **go to 25**) | **24. If yes, how many complaints of ADRs have you received in the last 4 weeks?** ……. |
| **25. Have you suspected an ADR in the last 4 weeks?**<br><br>[1] Yes [2] No (If no, **go to 28**) | **26. If yes, how many ADRs have you suspected in the last 4 weeks?** ............ |
| **27. Briefly describe the most recent suspected ADR you encountered providing information on patient age, drug involved & route of administration, outcome of ADR & its severity (mild, moderate, severe); e.t.c.**<br>……………………………………………<br>……………………………………………<br>……………………………………………<br>……………………………………………<br>…………………………………………… | |
| **28. Have you ever encountered a fatal ADR that might have led to a patient's death?**<br><br>[1] Yes [2] No | **29. Have you reported any suspected ADR in the last 12 months?**<br><br>[1] Yes [2] No (If No, **go to 35**) |
| **30. If yes, please indicate the period within which you reported the most recent suspected ADR**<br><br>[1] **4 weeks** [2] **5-8 weeks** [3] **9-12 weeks** [4] **4-6 mo** [5] **7-12 mo** | **31. To which authorities did you report the most recent of these ADRs?**<br>(*Tick all that apply*)<br>[1] National Drug Authority (NDA)<br>[2] AIDS Treatment Information Centre (ATIC)<br>[3] Drug Manufacturer<br>[4] Medical Superintendent<br>[5] District Director of Health Services (DDHS)<br>[6] Institutional Review Board (IRB)<br>[7] Other (specify)………………................ |
| **32. What motivated you to report the suspected ADR?**<br>……………………………………<br>……………………………………<br>…………………………………… | **33. Did you get any feedback about the ADR report(s) you submitted?**<br><br>[1] Yes [2] No |
| **34. Have you reported an ADR to the National Drug Authority in the past 12 months?**<br><br>[1] Yes [2] No | **35. Have you wanted to report an ADR in the past 12 months but did not have the ADR report form?**<br><br>[1] Yes [2] No |
| **36. Have you had an ADR suspicion in the past 12 months but did not fill the ADR report form even when you had it?**<br><br>[1] Yes [2] No | **37. Did you ever fill the ADR report form but failed to send it for any reason?**<br><br>[1] Yes [2] No (If no, **go to 39**) |

For peer review only

| | |
|---|---|
| **38. If yes, what was the reason(s) that you did not send the form on the most recent occasion?**<br>…………………………………....<br>…………………………………....<br>…………………………………....<br>………………………………….... | **39. Which of the following health workers are qualified to report adverse drug reactions?** (Tick all that apply)<br>[1] Medical doctors [4] Pharmacists<br>[2] Dentists [5] Clinical Officers<br>[3] Nurses |
| **40. Pharmacovigilance relates to a reporting system for adverse effects of medicines. Have you ever heard about Pharmacovigilance?**<br>[1] Yes [2] No (If no, **go to 42**) | **41. If yes, please state the source(s) of your information** (Tick all that apply)<br>[1] Books/Journals<br>[2] Internet/e-communication<br>[3] Trainings/Seminars/courses attended<br>[4] Television<br>[5] Outdoor adverts<br>[6] Professional colleague<br>[7] Others (Specify)…………………………. |
| **42. Are you aware of the existence of a National Pharmacovigilance Centre (NPC) in Uganda?**<br>[1] Yes [2] No (If no, **go to 44**) | **43. If yes, do you know where the NPC office is located?**<br>[1] Yes [2] No |
| **44. Have you ever seen the ADR form used for reporting ADRs to the NPC?**<br>[1] Yes [2] No (If no, **go to 48**) | **45. If yes, have you ever filled out the NPC ADR form?**<br>[1] Yes [2] No (If no, **go to 47**) |
| **46. Was the information on the NPC ADR form clear to you about what to report?**<br>[1] Yes [2] No | **47. Have you ever filled out any ADR form different from that of the NPC?**<br>[1] Yes [2] No |
| **48. Have you ever submitted an ADR report to the NPC?**<br>[1] Yes [2] No | **49. Do you know where to obtain the NPC ADR forms in this health facility?**<br>[1] Yes [2] No |
| **50. Do you know to whom to report ADRs in your health facility?**<br>[1] Yes [2] No (If no, **go to 52**) | **51. If yes, please specify in your health facility to whom you would report an ADR if you had to?**<br>…………………………………...<br>………………………………….... |
| **52. An ADR reporting system should;** (*Tick all that apply*)<br>[1] be compulsory<br>[2] be voluntary<br>[3] provide financial incentives to the reporter<br>[4] hide the identity of the prescriber<br>[5] hide the identity of the reporter | **53. Have you ever been trained on how to report ADRs with the ADR form?**<br>[1] Yes [2] No |

For peer review only

| [6] hide the identity of the patient | |
|---|---|
| **54. Please suggest possible ways of improving ADR reporting** ........................................ ........................................ | |

**Instructions**
In the left column are questions that will be the subject of your evaluation and in the right column is a gradual scale where you should mark with **X** the place along the scale where, according to your opinion, represents your degree of agreement with the text comment. The **extreme left** side indicates **total disagreement** while the **extreme right** indicates **total agreement**. Agreement increases as you move across from left to right
Please indicate whether you agree or disagree with the following statements
(1 = Strongly disagree; 2 = Slightly disagree; 3 = Neutral; 4 = Slightly agree; 5 = Strongly agree)

Strongly Disagree ←→ Strongly Agree

| | **Statement** | 1 | 2 | 3 | 4 | 5 |
|---|---|---|---|---|---|---|
| 55 | Serious ADRs are well documented by the time a drug is marketed | | | | | |
| 56 | It is nearly impossible to determine whether a drug is responsible for a particular adverse reaction | | | | | |
| 57 | I would only report an ADR if I was sure that it was related to the use of a particular drug | | | | | |
| 58 | The one case of an ADR that an individual health worker might see makes no significant contribution to medical knowledge | | | | | |
| 59 | I read articles about adverse drug reactions with interest | | | | | |
| 60 | I have a professional obligation to report ADRs | | | | | |
| 61 | Reporting ADRs puts my career at risk | | | | | |
| 62 | It is only necessary to report serious or unexpected ADRs | | | | | |
| 63 | I do not have time to complete an ADR report form | | | | | |
| 64 | I do not have the time to actively look for ADRs while at work | | | | | |
| 65 | I do not know how information reported in ADR form is used | | | | | |
| 66 | I talk with pharmaceutical companies about possible ADRs with their drugs | | | | | |
| 67 | I think that the best way to report ADRs is by publishing in medical literature | | | | | |
| 68 | I should be financially reimbursed for providing the ADR service | | | | | |
| 69 | I would be more likely to report ADRs if there were an easier method | | | | | |

For peer review only
